# CSF1R antagonism results in increased supraspinal infiltration in EAE

**DOI:** 10.1186/s12974-024-03063-1

**Published:** 2024-04-20

**Authors:** Marilyn Wang, Sofia E. Caryotakis, Glendalyn G. Smith, Alan V. Nguyen, David E. Pleasure, Athena M. Soulika

**Affiliations:** 1 Department of Dermatology, School of Medicine, University of California, Davis, Sacramento, CA USA; 2 Shriners Hospitals for Children, Northern California, Sacramento, CA USA; 3 Department of Neurology, School of Medicine, University of California, Davis, Sacramento, CA USA; 4 Present Address: University of California, San Francisco, San Francisco, CA USA; 5 Present Address: Sutro Biosciences, South San Francisco, CA USA

## Abstract

**Background:**

Colony stimulating factor 1 receptor (CSF1R) signaling is crucial for the maintenance and function of various myeloid subsets. CSF1R antagonism was previously shown to mitigate clinical severity in experimental autoimmune encephalomyelitis (EAE). The associated mechanisms are still not well delineated.

**Methods:**

To assess the effect of CSF1R signaling, we employed the CSF1R antagonist PLX5622 formulated in chow (PLX5622 diet, PD) and its control chow (control diet, CD). We examined the effect of PD in steady state and EAE by analyzing cells isolated from peripheral immune organs and from the CNS via flow cytometry. We determined CNS infiltration sites and assessed the extent of demyelination using immunohistochemistry of cerebella and spinal cords. Transcripts of genes associated with neuroinflammation were also analyzed in these tissues.

**Results:**

In addition to microglial depletion, PD treatment reduced dendritic cells and macrophages in peripheral immune organs, both during steady state and during EAE. Furthermore, CSF1R antagonism modulated numbers and relative frequencies of T effector cells both in the periphery and in the CNS during the early stages of the disease. Classical neurological symptoms were milder in PD compared to CD mice. Interestingly, a subset of PD mice developed atypical EAE symptoms. Unlike previous studies, we observed that the CNS of PD mice was infiltrated by increased numbers of peripheral immune cells compared to that of CD mice. Immunohistochemical analysis showed that CNS infiltrates in PD mice were mainly localized in the cerebellum while in CD mice infiltrates were primarily localized in the spinal cords during the onset of neurological deficits. Accordingly, during the same timepoint, cerebella of PD but not of CD mice had extensive demyelinating lesions, while spinal cords of CD but not of PD mice were heavily demyelinated.

**Conclusions:**

Our findings suggest that CSF1R activity modulates the cellular composition of immune cells both in the periphery and within the CNS, and affects lesion localization during the early EAE stages.

**Supplementary Information:**

The online version contains supplementary material available at 10.1186/s12974-024-03063-1.

## Introduction

Colony stimulating factor 1 receptor (CSF1R) is a tyrosine kinase receptor that promotes the development, survival, proliferation, migration, and differentiation of various myeloid cells such as monocytes, macrophages, dendritic cells (DCs), and osteoclasts [1, 2]. Its known cognate ligands are colony stimulating factor 1 (CSF1) and interleukin 34 (IL34). Although their expression patterns differ [3], and IL34 also signals via protein tyrosine phosphatase zeta (PTP-ζ) [4], these ligands induce relatively similar (albeit not identical) effects [5, 6].

Within the healthy central nervous system (CNS), CSF1R is expressed by microglia [5, 7, 8], and is necessary for their viability [9]. In addition, CSF1R is expressed by DCs [10, 11] and macrophages [12] in the choroid plexus and meninges. Although expression of CSF1R in neurons has been debated [5], it is now recognized that certain neurons express CSF1R in steady state, and CSF1R is upregulated in injury [13, 14].

The importance of CSF1R signaling in development is underlined by seminal studies showing that CSF1R deficiency and *Csf1*-null mutations lead to perinatal death [15]. Small molecule antagonists have been successfully and widely employed to elucidate the effects of CSF1R signaling in preclinical models [16–26]. These antagonists were originally developed to deplete tumor-associated macrophages, thus promoting a more pro-inflammatory and anti-tumor environment [27, 28]. In particular, PLX3397 (Plexxikon, Inc) is now FDA approved for the treatment of the rare and highly fatal tenosynovial giant cell tumors [29, 30]. PLX3397 was later shown to deplete microglia, and was used in neuroimmunological studies [9]. However, in addition to CSF1R, PLX3397 also targets c-KIT and FLT3 kinases [31], and has poor blood brain barrier (BBB) permeability [9, 23]. Other CSF1R antagonists, such as BLZ945 and PLX5622, were later developed, exhibiting improved potency and specificity [32]. PLX5622 is one of the most selective and potent (IC_50_ < 10nM) CSF1R antagonists, readily crosses the BBB, and is suitable for neuroimmunological studies [23, 33].

Both CSF1 and CSF1R are upregulated within and around demyelinating lesions of progressive multiple sclerosis (MS) patients compared to the white matter of non-MS controls [34]. CSF1R antagonism was previously shown to mitigate experimental autoimmune encephalomyelitis (EAE) severity by other groups [16, 17, 35], and this was initially attributed to microglia depletion [16, 34]. However, due to its broad expression, it is unlikely that the contribution of CSF1R to neuroinflammation is limited to microglia depletion. Indeed, thorough analysis of the effects of CSF1R antagonists has shown that other myeloid subsets are depleted both in steady state and in inflammation [17–20, 36]. Furthermore, even if CSF1R antagonism does not affect viability, it can affect cellular functions either directly or indirectly. For example, CSF1R antagonism impairs monocyte differentiation to macrophages [37, 38], and affects phagocytosis [18, 39]. Therefore, a characterization of multiple cell subsets is necessary to define the effects of CSF1R activity in neuroinflammation.

To test the effects of CSF1R antagonism in the initiation and progression of EAE, we employed PLX5622. Mice were analyzed both in steady state (i.e., treated with PLX5622 but not induced with EAE) and before and after the onset of EAE clinical symptoms. We show that in addition to microglia depletion, PLX5622 treatment modulated steady-state frequencies and/or numbers of various myeloid cells in the CNS, spleen, bone marrow, and skin. In agreement with other studies [16, 17, 35], we show that PLX5622 diminished EAE clinical severity. This was accompanied by decreased peripheral activation in the secondary lymphoid organs (SLOs) during the preclinical phase of EAE. Contrary to other studies, we found increased numbers of infiltrating leukocytes in the CNS of PLX5622-treated mice compared to controls. Among the CNS infiltrating cells, relative frequencies and numbers of inflammatory myeloid subsets were increased in the PLX5622 group compared to controls. Interestingly, CNS infiltrates were mainly localized in the cerebellum of PLX5622-treated mice, and mainly in the spinal cord of control mice. These data highlight an underappreciated effect of CSF1R in EAE, and suggest that CSF1R signaling affects the regional localization of inflammatory foci in neuroinflammation.

## Materials and methods

### Animals

C57BL/6 mice were obtained from Jackson Laboratory, and bred at the Mouse Biology Program at the University of California, Davis. Animal procedures were approved by the Institutional Animal Care and Use Committee of UC Davis. Animals were age- and sex-matched for each experiment.

### Compounds

PLX5622 (Plexxikon, Inc.) was formulated at 1200 mg/kg in AIN-76A chow by Research Diets, Inc. PLX5622 diet and AIN-76A control diet were given to mice ad libitum.

Rodent myelin oligodendrocyte glycoprotein peptide, amino acids 35–55 (MOG_35 − 55_), was obtained from Vivitide (now Biosynth).

### MOG_35 − 55_ EAE induction

Mice were fed PLX5622 diet or control diet for 7 days before EAE induction. Both male and female mice were used in this study. Age- (10–13 week-old) and sex-matched mice were EAE-induced using two subcutaneous flank injections of a total of 300 µg MOG_35 − 55_ in complete Freund’s adjuvant [incomplete Freund’s adjuvant (ThermoFisher) containing 5 mg/ml of heat-killed *Mycobacterium tuberculosis* (BD Difco Adjuvants)]. On days 0 and 2 after induction, mice also received intraperitoneal injections of 200 ng of pertussis toxin. Mice were weighed and assessed for clinical symptoms for classical EAE on a 5-point scale: 0 no detectable symptoms; 1 waddling gait, normal tail; 2 full limp tail, waddling gait; 3 hind limb paresis; 4 full hind limb paralysis; 5 death [40–43]. Half point scores were assigned when the severity of clinical symptoms were in between full-scale points.

### Single cell suspension preparation and flow cytometric analysis

Animals were anesthetized with an intraperitoneal injection of ketamine (150 mg/kg) and xylazine (16 mg/kg), and exsanguinated via transcardial perfusion using ice-cold PBS, as we have previously described [40, 43]. Following PBS perfusion, bone marrow or spleen and lymph nodes were passed through a 100 μm cell strainer in PBS. Red blood cells were lysed using ammonium-chloride-potassium (ACK) lysis buffer (Quality Biological Inc) and cells were resuspended in complete RPMI [(RPMI 1640 containing 10% FBS (Hyclone), 2 mM l-glutamine, 0.1 mM nonessential amino acids, 100 U penicillin-streptomycin, 1 mM sodium pyruvate (Gibco), and 50 µM 2-mercaptoethanol (Sigma)].

For T cell recall analysis, 300,000 spleen and lymph node cells/well were incubated in 96 well plates, in the presence or absence of 100 µg/ml MOG_35 − 55_ for 16 h in a total volume of 200 µL. All cells were then incubated in the presence of brefeldin A and monensin (Biolegend) for 5 h before immunostaining for flow cytometry analysis.

Pooled whole brain and whole spinal cord tissues were minced and incubated with 0.04 units of Liberase TL (Roche) and 10 µg of DNase I (Roche) in 5 ml PBS for 45 min at 37 °C. Shaved and depilated dorsal skin adjacent to the sites of immunization were minced and incubated with 0.4 U/ml Dispase II (Roche), for 2 h, at 37 °C. 1 mg/ml collagenase (Roche) was added and incubated for an additional 30 min [44, 45].

Immune cells were isolated using a 40/70% (v/v) Percoll gradient (GE Healthcare) and resuspended in cRPMI. For CNS infiltrating T cell analysis, cells were incubated in the presence or absence of 50 ng/ml phorbol 12-myristate 13-acetate (MilliporeSigma) and 750 ng/ml ionomycin (CalBioChem), as well as brefeldin A and monensin (Biolegend) for 3 h, and cells were immunostained. Live cells were detected using Zombie Red viability stain (ThermoFisher), after which cells were stained for extracellular markers, and fixed using 4% PFA at room temperature for 20 min before analysis. Cytokines were detected via intracellular staining using the BD Biosciences Cytofix/Cytoperm kit. FoxP3 was detected following cell fixation and nuclear permeabilization using the eBioscience FoxP3/Transcription factor buffer set. Cells were analyzed using the Attune NxT flow cytometer. Frequencies reported represented percentages of cell populations within their respective mother gates. Antibodies used are listed in Table 1.

**Table 1:** Antibodies used for flow cytometric analysis

Target	Clone	Manufacturer	Cat. number
CD4	RM4-5	BD	558107
CD8	53 − 6.7	BD	553031
CD11b	M1/70	Biolegend	191241
CD11b	M1/70	Invitrogen	48-0112-82
CD11b	M1/70	BD	552850
CD11c	HL3	Biolegend	550261
CD11c	HL3	BD	561241
CD11c	N418	Biolegend	117305
CD25	PC61.5	Invitrogen	17-0251-82
CD26	DPP-4	Biolegend	137803
CD45	RA3-6B2	Invitrogen	11-0452-82
CD45	30-F11	Biolegend	103130
CD88	20/70	Biolegend	135807
CD115	AFS98	Biolegend	135513
CD115	T38-320	BD	565249
FoxP3	FJK-15s	Invitrogen	12-5773-82
IFNγ	XMG1.2	BD	554413
IL17	TC11-18H10	BD	559502
Langerin	4C7	Biolegend	144206
Ly6C	HK1.4	Biolegend	128033
Ly6G	1A8	BD	551460
Ly6G	1A8	BD	560601
MHCII	M5/114.15.2	Biolegend	107652

### Evans blue analysis

Within 24 h of onset of typical EAE symptoms, mice were injected intravenously with 200 µL of 0.3% Evans blue dye (Sigma) dissolved in saline. Mice were anesthetized and perfused with PBS after 90 min [40]. Whole spinal cord and brain tissues were isolated and imaged immediately.

### Quantitative polymerase chain reaction (qPCR)

Lumbar spinal cords and cerebella were isolated at onset and acute EAE and stored in RNA*later* (Thermofisher Scientific). Tissues were homogenized in Qiazol (Qiagen), extracted using phenol-chloroform, and mRNA was purified using the RNeasy mini kit (Qiagen), according to the manufacturer’s instructions. cDNA was synthesized using the Quantitect Reverse Transcription kit (Qiagen) according to manufacturer’s instructions. qPCR was performed using an AriaMX real-time PCR system (Agilent) using SYBR Green Master Mix (Qiagen). Transcript expression levels were normalized using HSP90 [41]. Primers used in the study are listed in Table 2.

**Table 2:** List of primers used in the study

Gene name	NCBI Gene ID	Qiagen cat. number/ primer sequence (Fwd:Rev)	
*Hsp90*	NM_015516	QT01039864	
*Csf1r*	NM_007779	QT01055810	
*Csf2*	NM_009969	QT00251286	
*Csf3*	NM_009971	QT00105140	
*Il34*	NM_029646	QT00198142	
*Csf1*	NM_007778	QT01164324	
*Il1beta*	NM_008361	QT01048355	
*Ifng*	NM_008337	QT01038821	
*Il17a*	NM_010552	QT00103278	
*Tnf*	NM_013693	QT00104006	
*Il2ra*	NM_008367	QT00101108	
*Il12a*	NM_008351	QT01048334	
*Tgfb1*	NM_011577	QT00145250	
*Nos2*	NM_010927	CAGCTGGGCTGTACAAACCTT	CATTGGAAGTGAAGCGTTTCG
*Arg1*	NM_007482	QT00134288	
*Foxp3*	NM_054039	AGGAGCCGCAAGCTAAAAGC	TGCCTTCGTGCCCACTGT
*Rorgc*	NM_011281	CCGCTGAGAGGGCTTCAC	TGCAGGAGTAGGCCACATTACA
*Ccr2*	NM_009915	ATCCACGGCATACTATCAACATC	CAAGGCTCACCATCATCGTAG
*Ccr5*	NM_009917	GTCAGAACGGTCAACTTTGGG	GCCCAGAGTGATACAGATGTCAA
*Ccr6*	NM_009835	TCGTCCAGGCAACCAAATCCT	TCAGAGAACTTCCAGGTGAAGA
*Cd3e*	NM_007648.5	TGCCTCAGAAGCATGATAAGC	AAATGGACGCCGAACTTGGT
*Cd4*	NM_013488.3	TCCTAGCTGTCACTCAAGGGA	GTGTGGAAAATGAGGACTGCAT
*Cd8a*	NM_001081110.2	CCACGTTATCTTGTTGTGGGATG	GGGCTTGAGATGATGATGGAGA
*Cxcr2*	NM_009909	ATGCCCTCTATTCTGCCAGAT	GTGCTCCGGTTGTATAAGATGAC
*Cxcr3*	NM_009910	TATGGGGAAAACGAGAGCGAC	GCGCTGACTCAGTAGCACAG
*Ccl2*	NM_011333	QT00167832	
*Ccl4*	NM_013652	TTCCTGCTGTTTCTCTTACACCT	CTGTCTGCCTCTTTTGGTCAG
*Ccl6*	NM_009139	GCTGGCCTCATACAAGAAATGG	GCTTAGGCACCTCTGAACTCTC
*Ccl8*	NM_021443	TCTACGCAGTGCTTCTTTGCC	AAGGGGGATCTTCAGCTTTAGTA
*Ccl20*	NM_016960	QT02326394	
*Cxcl1*	NM_008176	CTGGGATTCACCTCAAGAACATC	CAGGGTCAAGGCAAGCCTC
*Cxcl9*	NM_008599	TCCTTTTGGGCATCATCTTCC	TTTGTAGTGGATCGTGCCTCG
*Cxcl10*	NM_021274	QT00093436	
*Cxcl11*	NM_011330	GAATCACCAACAACAGATGCAC	ATCCTGGACCCACTTCTTCTT
*Vcam1*	NM_011693	QT00128793	
*Itga4*	NM_010576	QT00121044	
*Icam1*	NM_010493	QT00155078	
*Itgal*	NM_001253874	QT01744085	

### Immunohistochemical analysis

Animals were anesthetized via intraperitoneal injection of ketamine and xylazine, and perfusion via cardiac puncture was performed using ice-cold PBS, as we previously described [40, 43]. CNS regions of interest were isolated, and tissues were post-fixed overnight in 4% PFA. After washing with PBS, tissues were cryoprotected in 30% sucrose for 3 days, and frozen in OCT.

Cerebellar and lumbar spinal cord cryosections (10 μm) were collected during EAE onset, dried for 1 h at room temperature, and blocked with 10% donkey serum for 1 h at room temperature. Following washes in PBS containing 0.1% Tween 20 (PBS-T), tissues were incubated overnight at 4 °C with primary antibodies against IBA1 (Wako Chemicals), CD3 (Santa Cruz Biotechnology), Ly6G (BD Pharmingen), and CD11b (BD Pharmingen). For MBP (BD Pharmingen) staining, slides were dipped in cold methanol for 30 min at -20 °C before initial washes and before blocking. Following washes in PBS-T, tissues were incubated with secondary antibodies conjugated with FITC, Rhodamine-X, or Cy5 (Jackson ImmunoResearch) for 1 h at room temperature, followed by DAPI counterstain. Tissues were mounted with Fluoromount-G (Electron Microscopy Sciences) and 20x images were acquired using the Nikon C2 scanning confocal microscope, stitched into whole tissue section images, and processed using the Nikon NIS-Elements AR software (version 4.60).

### Immunohistochemical quantification

Cells counts and fluorescence intensities were detected in whole tissue sections using Imaris software version 9.2 (Oxford Instruments). Cell counts (CD3+ or Ly6G+ cells) were calculated per mm^2^ of white matter area. Fluorescence intensities (for IBA1 and CD11b) were measured using Fiji ImageJ software (NIH) and shown as mean fluorescence intensity per pixel (MFI).

Demyelination was assessed via MBP immunostaining. Areas devoid of MBP immunoreactivity and total white matter areas were traced using Fiji ImageJ software (NIH); demyelination is shown as percentage of demyelinated area within the total white matter area.

In all immunohistochemical quantification, cerebellar and lumbar spinal cord tissues were matched per mouse and were collected during the onset of neurological deficits. At least two were sections averaged per tissue and 7–8 mice per group were analyzed.

### Sholl Analysis

Cerebella and lumbar spinal cord tissues were isolated during EAE onset, immunostained with IBA1 and scanned using a 60x oil objective on the Nikon C2 scanning confocal microscope. The simple neurite tracing tool on Fiji ImageJ software (NIH) was used to trace IBA1 + branches. Two images per section, 4–8 microglia per section, two sections per tissue and 6–7 mice were employed.

### Statistical analysis

To determine statistical significance for EAE clinical course, we employed the Mann-Whitney U test. Experiments were repeated at least 5 times with at least 4–6 mice per gender per group per timepoint. Significance for cumulative and peak disease scores, flow cytometry, cell number and MFI quantification, and Sholl analysis was calculated with two-tailed Student’s t-test. When not normally distributed, data were log transformed. Significance for transcript differences was calculated with one-way ANOVA. P values were statistically significant if < 0.05.

## Results

### CSF1R antagonism depletes microglia and modulates other myeloid cell populations in the steady state CNS

**Fig. 1:**
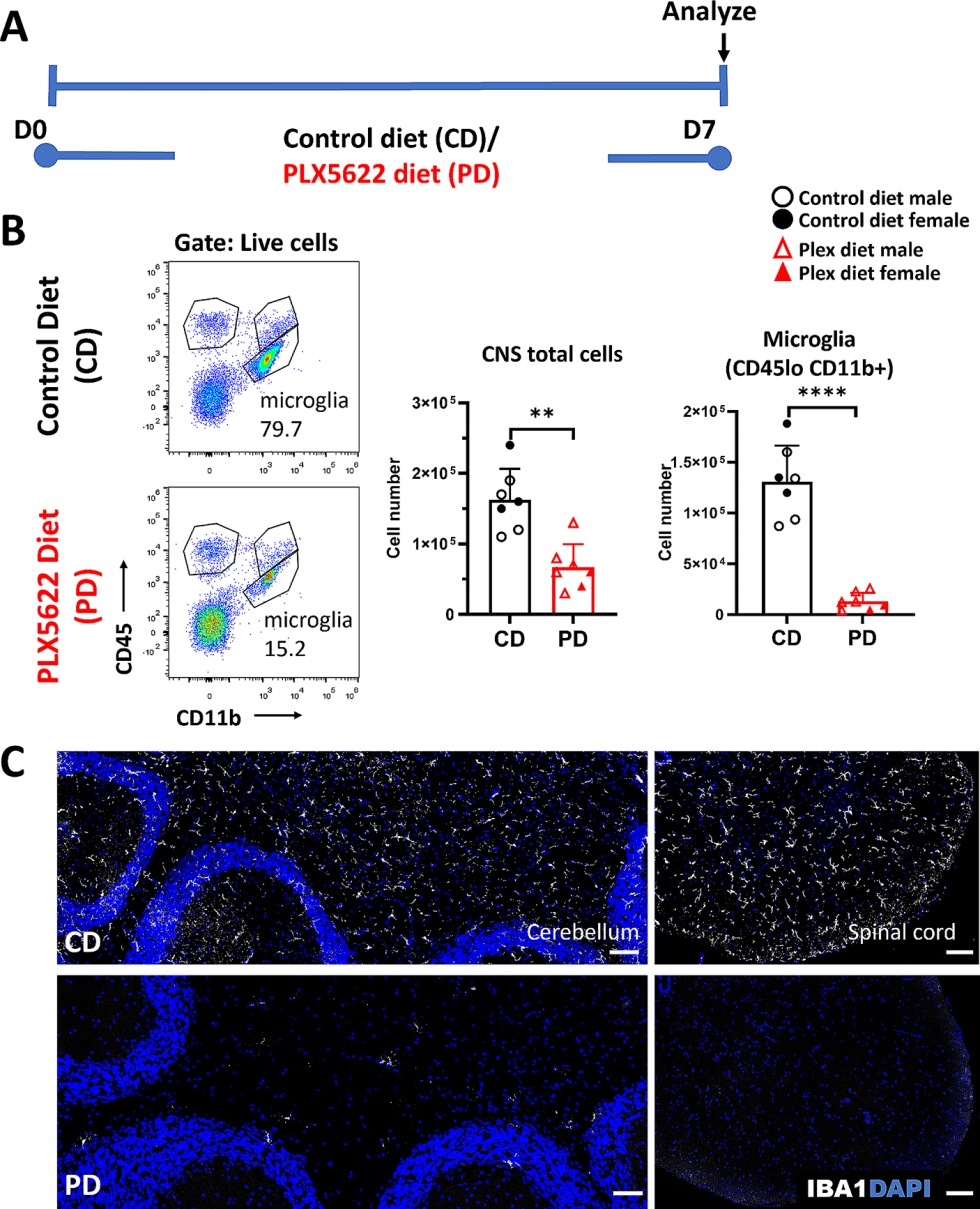
CSF1R antagonism depletes microglia at steady state. **A** Experimental schema depicting that healthy C57BL/6 mice were maintained on control diet (CD) or PLX5622 diet (PD) for seven days. Tissues were then collected and analyzed by flow cytometry and immunohistochemistry. **B** Flow cytometric analysis shows that both total CNS cells and CD11b+CD45lo (microglia) numbers are reduced in PD compared to CD mice in steady state. **C** Immunohistochemical staining of cerebellum (left) and spinal cord (right) tissues confirms robust IBA1+ cell depletion in PD mice. Scale bars represent 100 μm. Results are shown as means ± SD, *n* = 6–7; **p < 0.005, ****p < 0.00005

Mice were placed on either 1200 mg/kg PLX5622 diet (PD) or control diet (CD) for seven days (Fig. 1A; schema). Flow cytometric analysis of pooled spinal cord and brain (CNS) confirmed that PD mice showed statistically significantly decreased total CNS cell numbers compared with CD mice (CD: 1.63 × 10^5^ vs. PD: 6.71 × 10^4^, *p* = 0.0006) (Fig. 1B). In agreement with previous studies [9, 18], this was mainly due to a 90% decrease of microglial (CD45loCD11b+) numbers in the PD CNS compared to CD (CD: 1.31 × 10^5^ vs. PD: 1.33 × 10^4^; *p* = 0.00000019) (Fig. 1B). Microglia were effectively depleted in the cerebellum and the spinal cord (Fig. 1C and Additional file 1A).

Other immune cell populations are sparse in the steady-state CNS; small numbers of macrophages [46], monocytes [47], T cells, [48], and DCs [10] can be found in the meningeal spaces and the choroid plexus, while neutrophils are occasionally present and limited to the meningeal spaces [49]. PD mice had elevated frequencies and numbers of lymphocytes, neutrophils, and DCs compared to CD mice (Additional file 1B). This analysis, however, is limited by the small number of non-microglia immune cells in the steady-state CNS.

### CSF1R antagonism reshapes immune cell populations in peripheral tissues in steady state

**Fig. 2:**
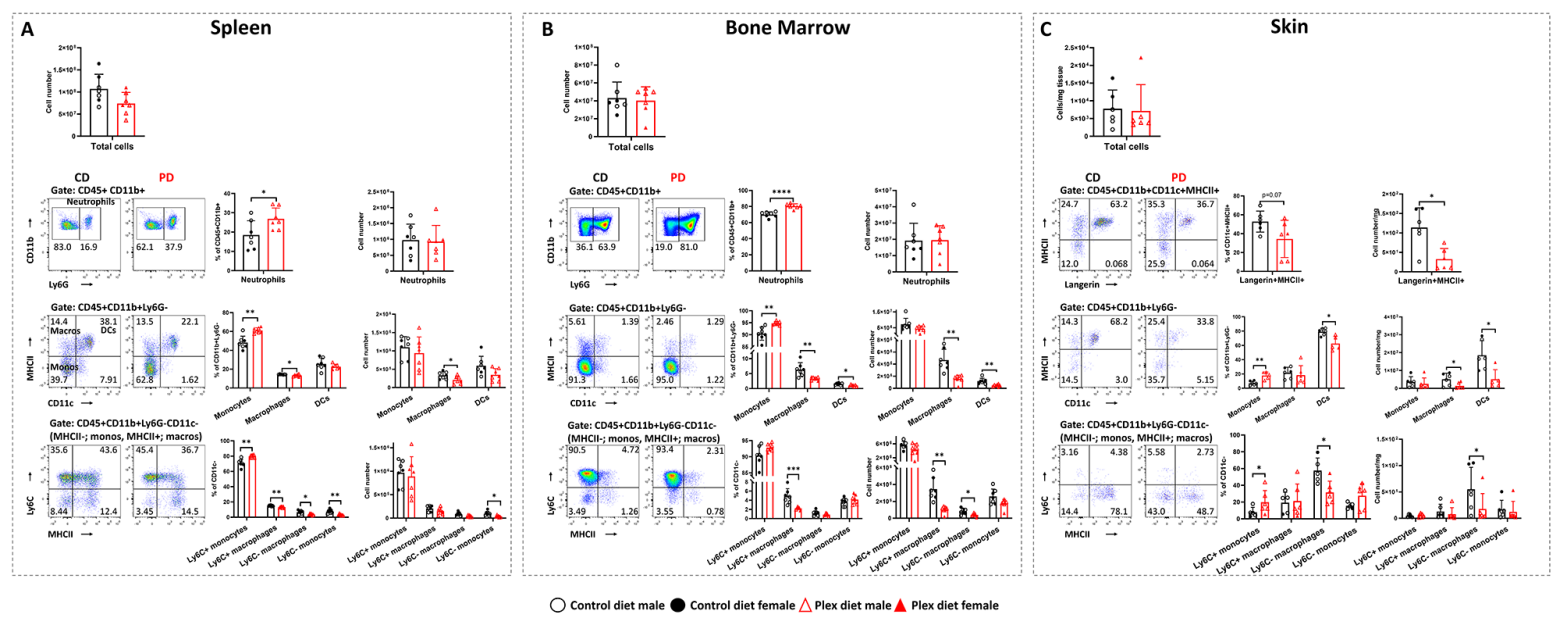
Effect of PLX5622 on myeloid cell subsets in peripheral immune tissues in steady state. Flow cytometric analysis of single cells isolated from spleen, bone marrow, and skin in CD and PD mice. Neutrophils (CD45+CD11b+Ly6G+), monocytes (monos; CD45+CD11b+CD11c-Ly6G-MHCII-Ly6C+), macrophages (macros; CD45+CD11b+CD11c-Ly6G-MHCII+) and dendritic cells (DCs; CD45+CD11b+CD11c+MHCII+) were analyzed in **A** spleen and **B** bone marrow. Monocytes and macrophage subsets were further classified as Ly6C+ (inflammatory) or Ly6C- (non-inflammatory). **C** In skin, dendritic cells (CD45+CD11b+CD11c+MHCII+), langerin + cells (CD45+CD11b+Ly6G-CD11c+MHCII+langerin+; includes Langerhans cells in the epidermis and langerin+ DCs in the dermis), and monocyte/macrophage populations were analyzed. Monocytes and macrophage subsets were further classified as Ly6C+ or Ly6C-. Representative flow plots are shown. Both male and female mice were employed. Results are shown as means ± SD, *n* = 6–7; *p < 0.05, **p < 0.005, ****p < 0.00005

Compared to CD, PD mice showed no statistically significant differences in total cell numbers neither in the spleen (CD: 1.07 × 10^8^ vs. PD: 7.37 × 10^7^; *p* = 0.06) (Fig. 2A) nor the bone marrow (BM) (CD: 4.13 × 10^7^ vs. PD: 4.03 × 10^7^; *p* = 0.8) (Fig. 2B). PD increased frequencies of neutrophils (CD45+CD11b+Ly6G+) in both the spleen and the BM, but not their absolute numbers. This suggests that the changes in neutrophil frequencies are due to a non-neutrophil cell population depletion.

PD increased total monocyte (CD45+CD11b+Ly6G-CD11c-MHCII-) frequencies and reduced total macrophage (CD45+CD11b+Ly6G-CD11c-MHCII+) frequencies and numbers both in the spleen and in the BM compared to CD (Fig. 2A and B). Frequencies of Ly6C+ monocytes increased, while Ly6C- monocytes and Ly6C+ and Ly6C- macrophages decreased in the spleens of PD compared to CD group (Fig. 2A). BM DC (CD45hiCD11b+Ly6G-CD11c+MHCII+) frequencies and numbers decreased in PD compared to CD mice (Fig. 2B). Additionally, the frequencies of Ly6C+ macrophages, and the numbers of Ly6C+ and Ly6C- macrophages decreased in the BM of the PD group. All of these are in agreement with a previous study by Lei et al. [18]. However, unlike Lei et al. [18], we did not observe significant differences in CD4+ T and CD8+ T cells neither in the spleen nor in the BM between the two groups (data not shown). This may be due to the shorter period of PD treatment in our study (1 week) versus the study by Lei et al. (3 weeks).

Analysis of healthy caudal dorsal skin (pooled epidermis and dermis) showed that CSF1R antagonism did not change the total number of cells per mg of tissue (Fig. 2C). PD, however, diminished total macrophages (CD45+CD11b+Ly6G-CD11c-MHCII+) and langerin+ cells (CD45+CD11b+Ly6G-CD11c+MHCII+langerin+), a population which includes Langerhans cells in the epidermis and langerin+ DCs in the dermis (Fig. 2C). This is in agreement with studies showing that CSF1R signaling is mandatory for Langerhans cells’ viability [50–52]. Frequencies of total monocytes and Ly6C+ monocytes increased in PD skin, but their numbers did not change between the groups. Furthermore, the numbers of total DCs and of Ly6C- macrophages were decreased in the PD group (Fig. 2C).

### CSF1R antagonism ameliorates EAE clinical course

**Fig. 3:**
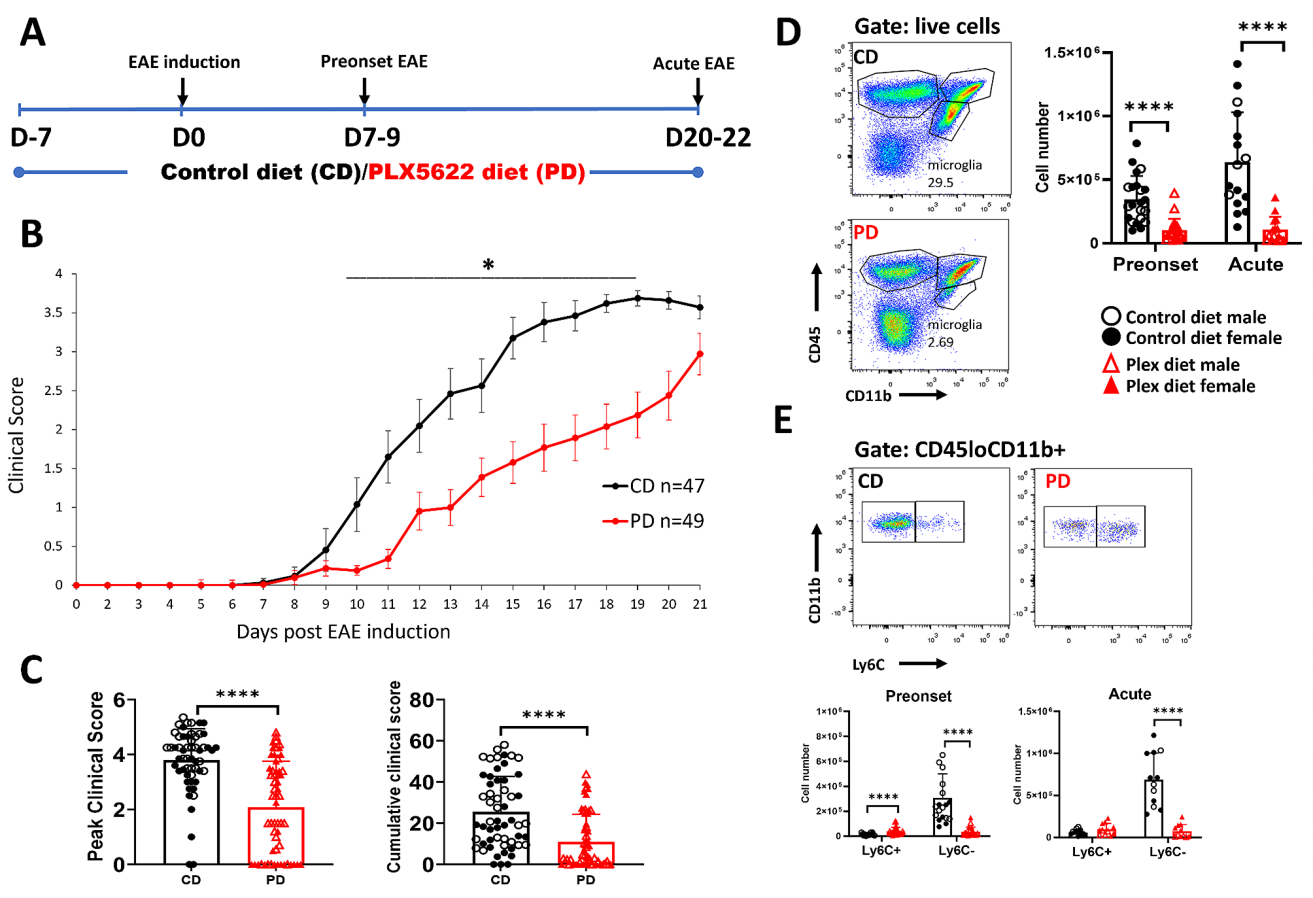
CSF1R antagonism ameliorates EAE clinical severity. **A** Schema of experimental design. **B** Classical EAE neurological symptoms onset was delayed, and severity was milder in PD compared to CD mice. **C** Peak and cumulative EAE scores were lower in PD compared to CD mice. **D** Microglia (CD45loCD11b+) are depleted in pooled brain and spinal cord tissues of PD mice in preclinical and acute EAE. **E** Analysis of Ly6C expression in CD45loCD11b+ cells shows that CD45loCD11b + Ly6C- cells were preferentially depleted in the CNS of PD mice. Representative flow plots are shown. Both male and female mice were employed. Results are shown as means ± SD; *n* = 44–47 CD, 40–49 PD; *p < 0.05, ****p < 0.0005

To examine the role of CSF1R signaling in the onset and progression of EAE, age- and sex-matched mice were placed on CD or PD for 7 days before EAE induction and maintained on their respective diets up to the end of the experiment (Fig. 3A; schema).

In agreement with previous studies [17, 35], PD mice exhibited delayed disease onset and milder severity of classical EAE clinical symptoms (Fig. 3B, Additional file 2), and lower peak (CD: 3.8 ± 1.1 vs. PD: 2.1 ± 1.7; *p* < 0.0001) and cumulative disease scores (CD: 25.4 ± 17.3 vs. PD: 10.9 ± 13.4; *p* < 0.0001), compared to the CD group (Fig. 3C).

Interestingly, 35% of PD mice exhibited atypical EAE neurological deficits such as seizures, ataxia, repetitive axial rotation, and anxiety and fear responses, such as freezing and strong startle reflexes; these mice exhibited no or very mild classical EAE clinical scores. Atypical EAE symptoms were never observed in CD mice. Unexpectedly, 60% of PD mice with atypical EAE (corresponding to 21% of total PD mice employed) died prior to development of conventional EAE symptoms, and before day 14 post EAE induction (pi) (not shown). PD mice that died prior to development of severe conventional EAE were not included in subsequent clinical scoring analysis. The rest of PD mice later also developed classical EAE deficits and none of these mice died as a result of EAE. We observed no deaths in the CD group.

No differences in EAE clinical course were noted between male and female mice in either group.

Flow cytometric analysis of pooled brain and spinal cord tissues (per mouse) before clinical EAE symptoms appear (pre-onset; days 7–9 pi) showed that 70% of CD45loCD11b+ cells (a population that is mostly microglia) were depleted in PD mice (CD: 3.46 × 10^5^ vs. PD: 1.03 × 10^5^; *p* = 0.0000015) (Fig. 3D). Further analysis showed that the depleted population was predominantly CD45loCD11b + Ly6C-, a population that has been suggested to denote mostly microglia, while CD45loCD11b+Ly6C+ denotes mostly infiltrating macrophages [53] (Fig. 3E).

### CSF1R antagonism diminishes peripheral activation in secondary lymphoid organs in EAE

To determine whether the milder EAE course in PD mice was due to impaired immune responses in the periphery, we employed flow cytometry to analyze single cell suspensions of pooled spleen and draining lymph nodes (herein referred to as the secondary lymphoid organs, SLOs), before clinical EAE symptoms appear (day 7–9 pi).

**Fig. 4:**
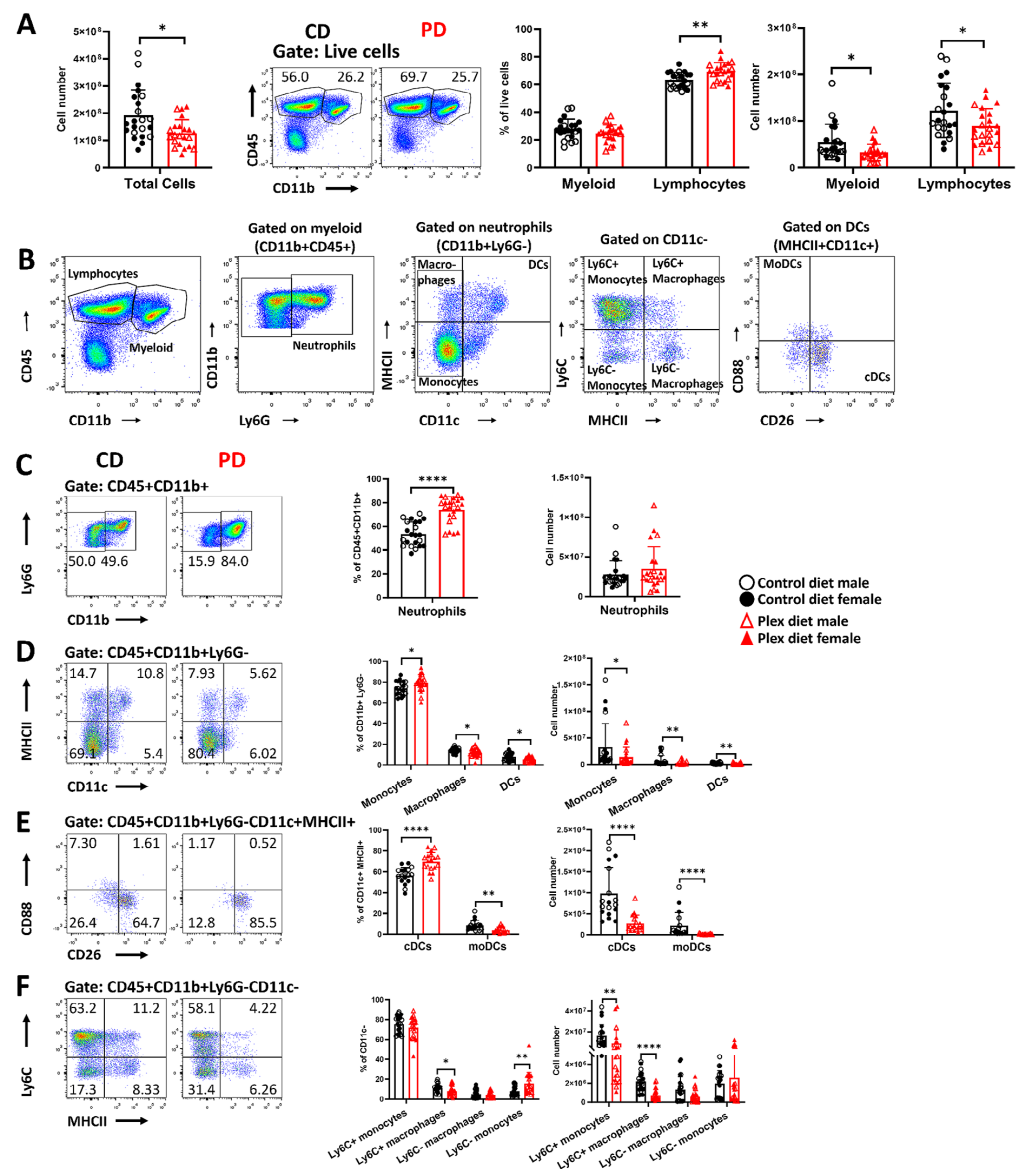
PD depletes macrophages and DCs in secondary lymphoid organs (SLOs) in preclinical EAE. Pooled spleen and draining lymph nodes (SLOs) were analyzed before clinical symptom onset (preonset EAE). **A** Numbers of total cells, and of myeloid (CD45+CD11b+) and lymphocytic (CD45+CD11b-) cells were lower in the SLOs of PD compared to those of CD mice. **B** Gating strategy for flow cytometry analysis of SLO myeloid cells in EAE. **C** Neutrophil (CD45+CD11b+Ly6G+) frequencies were increased but not their numbers in PD mice compared to CD mice. **D** Frequencies of all monocytes (CD45+CD11b+Ly6G-CD11c-MHCII-) were increased but their numbers were decreased in SLOs of PD compared to those of CD mice. Both frequencies and numbers of total DCs (CD45+CD11b+Ly6G-CD11c+MHCII+), and of total macrophages (CD45+CD11b+Ly6G-CD11c-MHCII+) were decreased in PD compared to CD. **E** Frequencies of classical DCs (cDCs; CD45+CD11b+Ly6G-CD11c+MHCII+CD26+CD88-) were increased, and those of monocyte-derived DCs (moDCs; CD45+CD11b+Ly6G-CD11c+MHCII+CD26-CD88+) were decreased but the absolute numbers of both subsets were decreased in the PD SLOs compared to CD. **F** Ly6C+ monocyte and macrophage subsets (CD45+CD11b+Ly6G-CD11c-MHCII+Ly6C+) were decreased in the PD SLOs compared to those of CD mice. Representative flow plots are shown. Both male and female mice were employed. Data are shown as means ± SD, *n* = 22; *p < 0.05, **p < 0.005, ****p < 0.00005

Absolute numbers of total cells in the SLOs of PD mice were reduced compared to CD mice (CD: 1.93 × 10^8^ ± 9.28 × 10^7^ vs. PD: 1.27 × 10^8^ ± 5.02 × 10^7^; *p* = 0.006), and this was associated with statistically significant reductions of both myeloid and lymphoid cell numbers (Fig. 4A). We further analyzed myeloid cell subsets using the gating strategy shown in Fig. 4B. CSF1R antagonism increased neutrophil frequencies, but not their absolute numbers (Fig. 4C). PD decreased total macrophage and DC frequencies and absolute numbers (Fig. 4D). Although the frequencies of classical DCs (cDCs; CD45+CD11b+Ly6G-CD11c+MHCII+CD26+) increased while those of monocyte-derived DCs (moDCs; CD45+CD11b+Ly6G-CD11c+MHCII+CD88+) decreased, the absolute numbers of both these subsets decreased (Fig. 4E). CSF1R antagonism statistically significantly decreased the numbers of Ly6C+ (inflammatory) monocytes [54] and macrophages in PD mice (Fig. 4F).

**Fig. 5:**
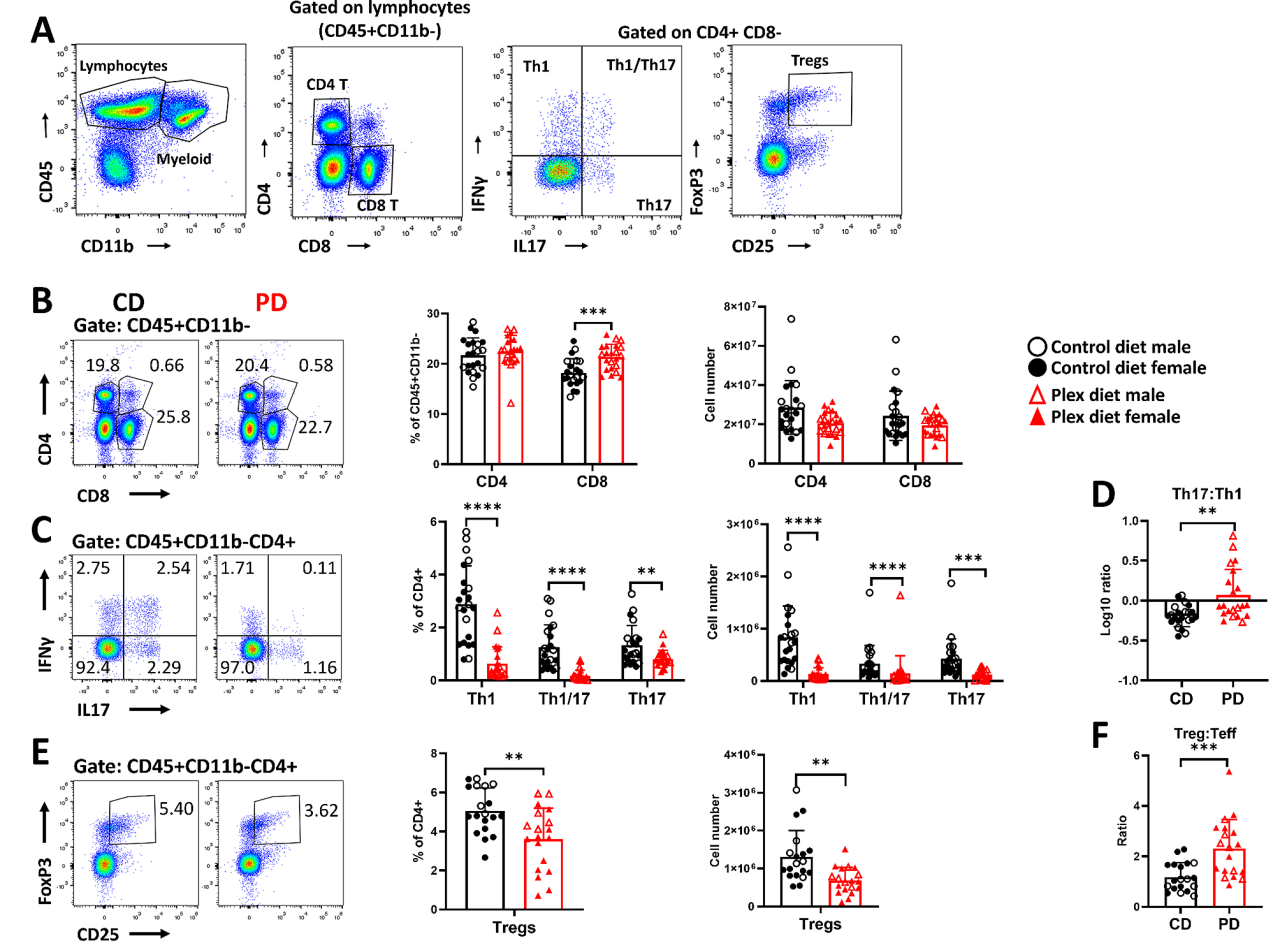
Impaired T effector cell generation in the SLOs of PD mice in preclinical EAE. **A** Gating strategy for flow cytometry analysis of T cell subsets in the EAE SLOs **B** Frequencies and numbers of CD4+ and CD8+ T cells in the SLOs of CD and PD mice. **C** Frequencies and numbers of MOG-specific CD4 T cell (CD45+CD11b-CD4+) subsets Th1 (IFNγ+), Th17 (IL17+), Th1/17 (IFNγ+IL17+) were decreased in the PD compared to CD SLOs. **D** Log10 ratios of Th17:Th1 were increased in the SLOs of PD compared to CD. **E** Both frequencies and absolute numbers of Tregs (CD25+FoxP3+) were decreased in PD SLOs compared to CD. **F** Ratios of Treg:Teff were increased in PD SLOs compared to CD. Representative flow plots are shown. Both male and female mice were employed. Data are shown as means ± SD, *n* = 22; **p < 0.005, ***p < 0.0005, ****p < 0.00005.

Analysis of lymphocytic subsets (gating strategy shown in Fig. 5A) showed that there were no differences in total CD4+ T cells between the two groups (Fig. 5B). PD, however, decreased frequencies and numbers of MOG-specific T effectors (Teff: Th1, Th17, and Th1/17) (Fig. 5C). Interestingly, the log10 ratio of Th17:Th1 was increased in PD compared to CD mice. This ratio was greater than 1 in PD and lower than 1 in CD mice (Fig. 5D). MOG-specific T regulatory cells (Tregs: CD4+CD25+Foxp3+) were decreased (Fig. 5E) while the ratio of MOG-specific Tregs to Teffs was elevated in the SLOs of PD compared to CD mice during preclinical EAE (Fig. 5F).

During later stages of the disease (acute EAE; day 20–22 pi) the frequencies and absolute numbers of neutrophils, total monocytes, and Ly6C + monocytes were elevated in PD compared to CD mice (Additional file 3A, B). There were no differences in DCs (Additional file 3B) and T cell (Additional file 3B) populations in the SLOs during this timepoint.

### CSF1R antagonism depletes myeloid cell populations in the bone marrow and skin in EAE

To determine if immune cell frequencies and numbers were affected by CSF1R antagonism in tissues other than the SLOs, we also analyzed bone marrow and dorsal skin (adjacent to the site of immunization) during preclinical and acute EAE.

There were no differences in the absolute numbers of total bone marrow cells, neither before the onset nor during acute EAE (Additional file 4). Neutrophil frequencies but not numbers were elevated, while DC and macrophage numbers were diminished in the PD bone marrow during preclinical EAE compared to controls (Additional file 4A). Additionally, Ly6C + monocytes and Ly6C+ and Ly6C- macrophages were decreased in the preclinical phase (Additional file 4A), but not during acute EAE (Additional file 4B).

In the skin, neutrophil frequencies were increased in preclinical EAE (Additional file 5A). Langerin+ cells, total macrophages, and Ly6C+ inflammatory macrophages were reduced in PD compared to CD mice, both during the preclinical phase and acute disease (Additional file 5A and B). DCs, and particularly skin langerin+ DCs, are recruited into the SLOs and are powerful antigen presenting cells [52], thus reduction in these cell types may contribute to the priming impairments in PD mice. Total monocytes were increased only in acute EAE (Additional file 5B). Immunohistochemical analysis confirmed that the skin of PD mice contained fewer CD11b+IBA1+ macrophages (Additional file 5C).

### CSF1R antagonism increases CNS infiltration by peripheral immune cells

Single cell suspensions from pooled brain and spinal cord tissues (per mouse) were analyzed by flow cytometry before clinical symptom onset or during acute EAE.

**Fig. 6:**
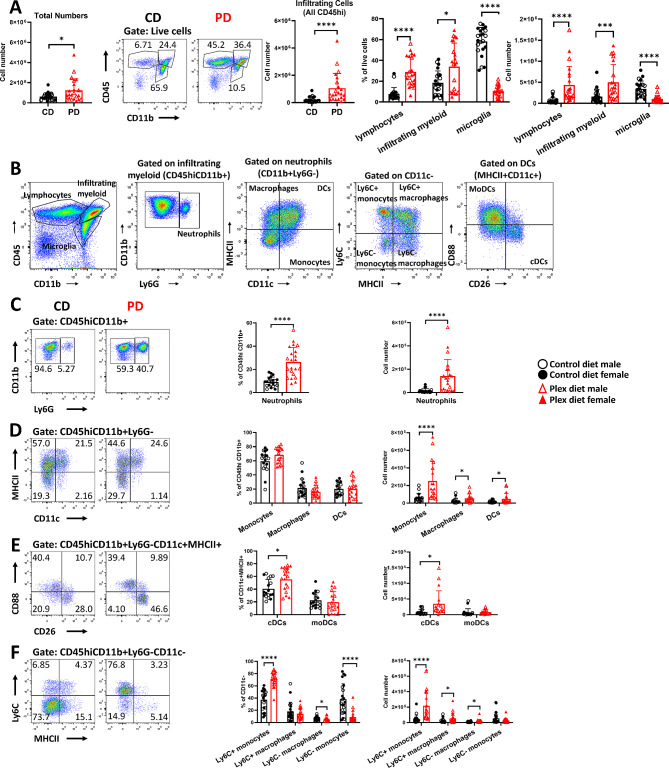
CSF1R antagonism increases CNS infiltration by myeloid cells before neurological symptoms appear in EAE. Single cells isolated from pooled brain and spinal cord tissues were analyzed by flow cytometry for myeloid subsets before onset of neurological deficits (preonset) **A** Numbers of total cells, and of infiltrating immune cells (CD45hi), which include myeloid cells (CD45hiCD11b+) and lymphocytes (CD45hiCD11b-) were increased while microglia were decreased in the CNS of PD compared to CD mice in preclinical EAE. **B** Gating strategy for flow cytometric analysis of myeloid cells in the CNS. **C** Frequencies and absolute numbers of neutrophils (CD45hiCD11b+Ly6G+) were increased in the CNS of PD mice with during preonset EAE compared with CD mice. **D** No differences in the frequencies of total monocytes (CD45hiCD11b+Ly6G-CD11c-MHCII-), total macrophages (CD45hiCD11b+Ly6G-CD11c-MHCII+) and total DCs (CD45hiCD11b+Ly6G-CD11c+MHCII+) were detected but their numbers were increased in the CNS of PD compared to CD. **E** Frequencies and numbers of cDCs (CD45hiCD11b+Ly6G-CD11c+MHCII+CD26+CD88-), but not of moDCs (CD45hiCD11b+Ly6G-CD11c+MHCII+CD26+CD88+) were increased in the CNS of PD compared to CD mice. **F** Numbers of Ly6C+ monocytes, and of both Ly6C+ and Ly6C- macrophages were increased in the CNS of PD compared to CD mice in preclinical EAE. Representative flow plots are shown. Both male and female mice were employed. Data are shown as means ± SD, *n* = 22; *p < 0.05, ***p < 0.0005, ****p < 0.00005

Despite efficient microglial depletion, the absolute numbers of total CNS cells before neurological deficits onset were higher in PD compared to CD mice (CD: 6.17 × 10^5^ ± 3.63 × 10^5^ vs. PD: 12.5 × 10^5^ ± 11.4 × 10^5^; *p* = 0.02) (Fig. 6A). This was due to increased numbers of infiltrating leukocytes (CD45hi) in the CNS of PD compared to CD mice (CD: 2.23 × 10^5^ ± 2.04 × 10^5^ vs. PD: 10.6 × 10^5^ ± 10.8 × 10^5^; *p* = 0.001) (Fig. 6A). Both infiltrating myeloid and lymphocyte subsets increased in the CNS of PD compared to CD mice (Fig. 6A).

We further analyzed the CNS infiltrating myeloid cells (gating strategy shown in Fig. 6B). Frequencies and numbers of neutrophils were elevated in the CNS of PD mice compared to CD mice (Fig. 6C). Although there were no differences in frequencies, numbers of total DCs, monocytes, and macrophages were elevated in the CNS of PD mice (Fig. 6D). Both frequencies and numbers of cDCs and Ly6C+ monocytes and numbers of Ly6C+ and Ly6C- macrophages were elevated in PD mice (Fig. 6E and F).

**Fig. 7:**
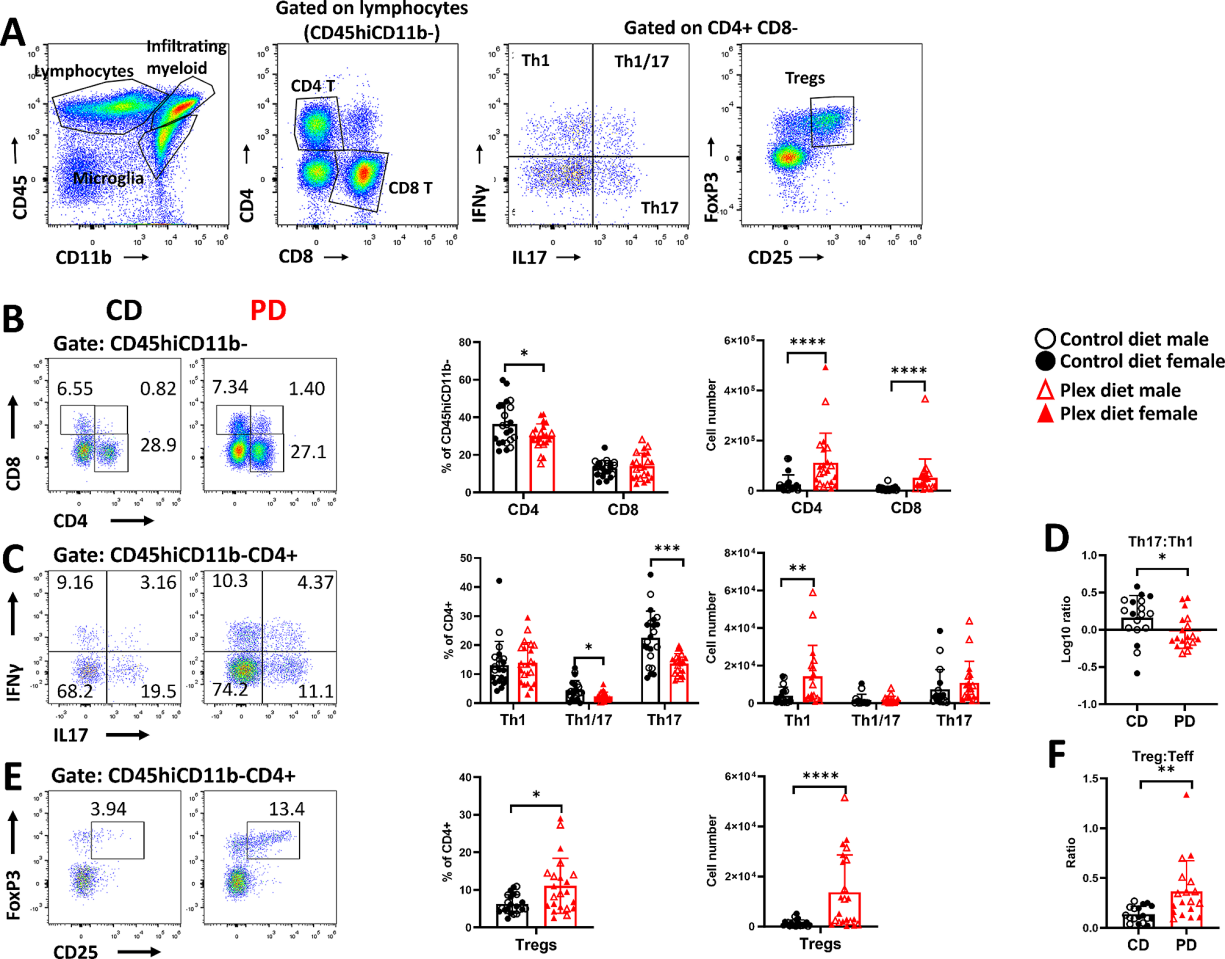
CSF1R antagonism increases CNS infiltration by lymphocytes before neurological symptoms appear in EAE. Single cells isolated from pooled brain and spinal cord tissues were analyzed by flow cytometry for T cell subsets before onset of neurological deficits (preonset). **A** Gating strategy for flow cytometric analysis of T cells in the EAE CNS. **B** Frequencies of both CD4+, but not of CD8+ T cells decreased, but the numbers of both subsets increased in the CNS of PD compared to CD mice. **C** Frequencies of Th1 cells were not different between the groups, but their absolute numbers were increased in the CNS of PD compared with CD. Although the frequencies of Th1/17 and Th17 cells were decreased their absolute numbers were not different in the CNS of PD compared to CD mice. **D** Log10 Th17:Th1 ratios were below 1 in the CNS of PD mice, while in CD they were greater than 1. **E** Both frequencies and numbers of Tregs were increased in the CNS of PD compared to CD mice. **F** Treg:Teff ratios were increased in the CNS of PD mice compared to CD before EAE onset. Representative flow plots are shown. Both male and female mice were employed. Data are shown as means ± SD, *n* = 22; *p < 0.05, **p < 0.005, ***p < 0.0005

CNS infiltrating lymphocytes (CD45hiCD11b-) were further analyzed as shown in Fig. 7A. The relative frequencies of CD4 + T cells were lower in PD compared to CD mice in preclinical EAE (Fig. 7B). However, and as a result of increased overall infiltration (Fig. 6A), the absolute numbers of both CD4 + T and CD8 + T cells were increased in PD mice (Fig. 7B). The relative frequencies of Th1 cells were not different between the groups, while those of Th17 and Th1/17 subsets were decreased in the CNS of PD compared to CD mice (Fig. 7C). However, the numbers of Th1 cells were elevated, while there were no changes in Th17 and Th1/17 numbers in PD compared to CD mice (Fig. 7C). This resulted in lower log10 ratios of Th17:Th1 in the CNS of PD compared to CD mice during preclinical EAE (Fig. 7D). These ratios were greater than 1 in the CD and lower than 1 in the PD mice (Fig. 7D).

Frequencies and absolute numbers of Tregs were increased (Fig. 7E) and accordingly, Treg:Teff ratios were significantly higher in preclinical EAE in the CNS of PD compared to CD mice (Fig. 7F).

**Fig. 8:**
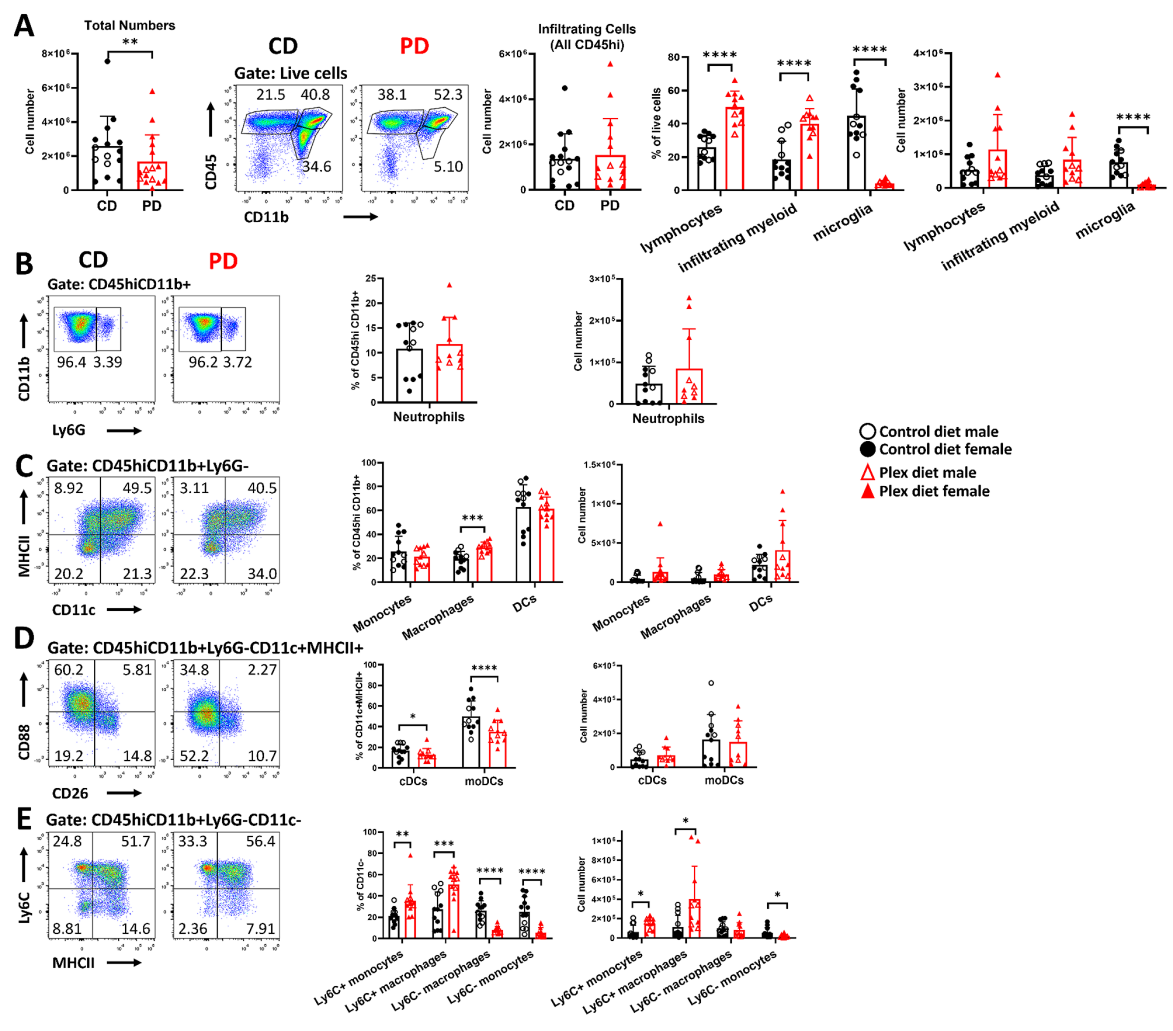
Only mild differences in the myeloid cell populations persist in the CNS of PD mice in acute EAE. Single cells isolated from pooled brain and spinal cord tissues were analyzed by flow cytometry during acute EAE. **A** Numbers of total cells were lower in the PD CNS compared to CD. There were no differences in the numbers of total infiltrating cells (CD45hi). Although the frequencies of myeloid cells (CD45hiCD11b+) and lymphocytes (CD45hiCD11b-) remained higher in PD compared to CD mice, their numbers were not different between the groups in acute EAE. Microglia frequencies and numbers remained low in PD compared to CD mice. **B** No differences in frequencies and absolute numbers of neutrophils were detected between the groups at this timepoint. **C** Although the frequencies of total macrophages (CD45hiCD11b+Ly6G-CD11c-MHCII+) were increased in the PD mice, there were no differences in absolute numbers. No differences were detected in total monocytes (CD45hiCD11b+Ly6G-CD11c-MHCII-) and total DCs (CD45hiCD11b+Ly6G-CD11c+MHCII+) between the groups. **D** Frequencies of cDCs and moDCs decreased, but their absolute numbers were not different in PD compared to CD. **E** Frequencies of Ly6C+ monocytes and macrophages were increased while Ly6C- monocytes and macrophages were decreased in the CNS of PD compared to CD mice during acute EAE. Representative flow plots are shown. Both male and female mice were employed. Data are shown as means ± SD, *n* = 12; *p < 0.05, **p < 0.005, ***p < 0.0005, ****p < 0.00005

**Fig. 9:**
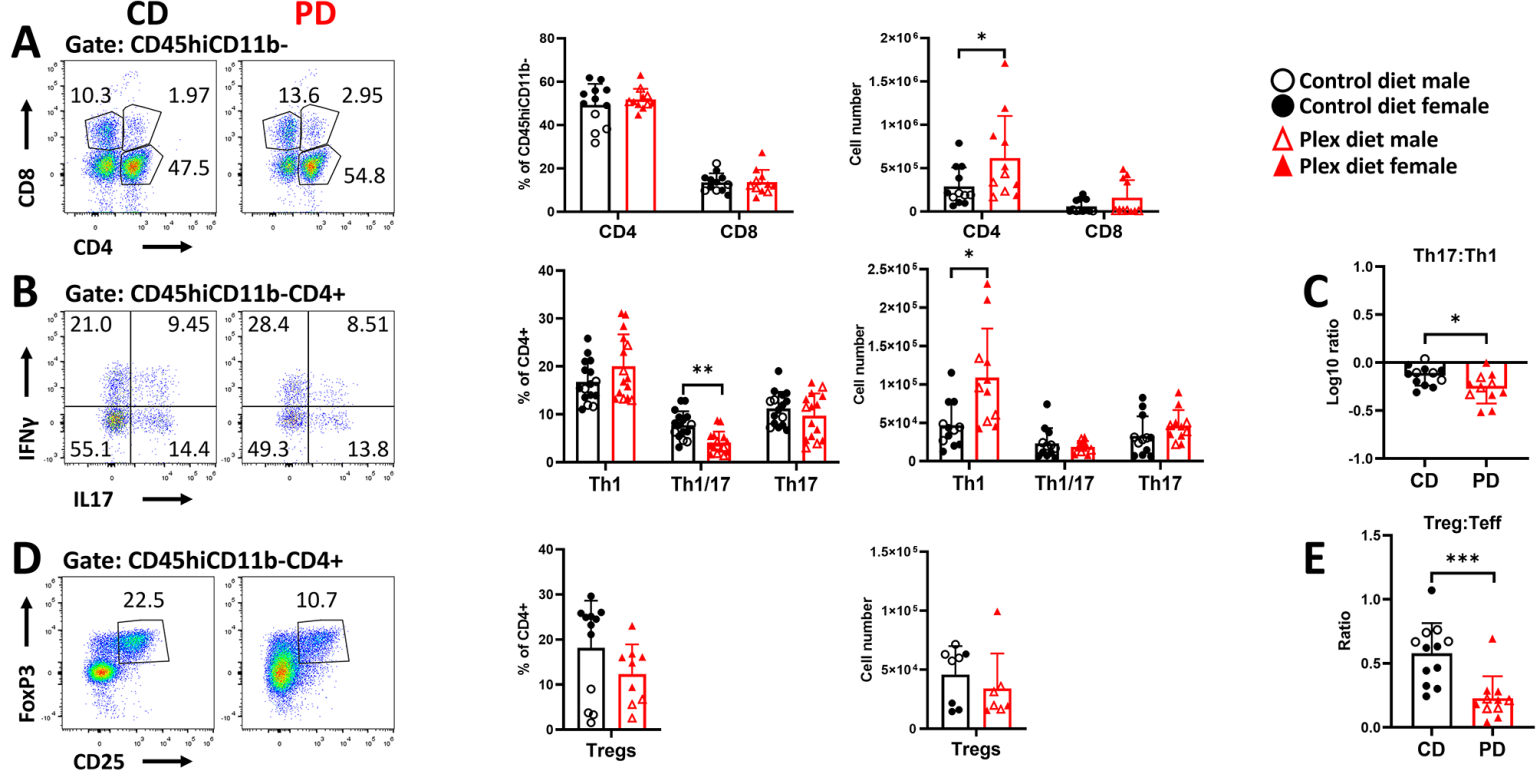
Mild differences in T cell populations persist in the CNS of PD mice during acute EAE. Single cells isolated from pooled brain and spinal cord tissues were analyzed by flow cytometry during acute EAE. **A** Although there were no differences in the CD4+ and CD8 + T cell frequencies, the numbers of CD4+ T cells were increased in the CNS of PD mice compared to CD. **B** Only numbers of Th1 cells remained elevated during acute EAE within the CNS of PD compared to CD mice. **C** Log10 Th17:Th1 ratios were below 1 for both groups and were significantly lower in the CNS of PD compared to CD mice. **D** There were no differences in the Treg populations between the groups. **E** Treg:Teff ratios were decreased in the CNS of PD compared to CD group. Representative flow plots are shown. Both male and female mice were employed. Data are shown as means ± SD, *n* = 12; *p < 0.05, **p < 0.005, ***p < 0.0005

During acute EAE (day 20–22 pi), the numbers of total cells in the CNS were decreased in the PD mice (Fig. 8A). There were no statistically significant differences between the two groups in the numbers of infiltrating leukocytes (CD45hi cells) (CD: 1.37 × 10^6^ ± 1.1 × 10^6^ vs. PD: 1.54 × 10^6^ ± 1.60 × 10^6^; *p* = 0.74), suggesting that the decrease in total cells is due to microglia depletion (Fig. 8A). At this timepoint, there were no differences in neutrophils and monocytes (Fig. 8B and C). Although the frequencies of both cDCs and moDCs were now reduced, their numbers were not different in PD compared to CD mice (Fig. 8D). The numbers of Ly6C+ inflammatory monocytes and macrophages were increased, while those of Ly6C- monocytes were decreased (Fig. 8E). Numbers of total CD4+ T cells and Th1 cells remained elevated in the CNS of the PD compared to CD group (Fig. 9A and B). The log10 ratio of Th17:Th1 remained decreased in the PD compared to CD group, and these ratios were lower than 1 in both groups during this timepoint (Fig. 9C).

Unlike the preclinical phase, Treg populations were now similar between the groups (Fig. 9D), and the Treg:Teff ratios were lower in the CNS of PD compared to CD mice (Fig. 9E).

These suggest that the CNS of PD mice is characterized by more robust infiltration, initiating during the preclinical EAE phase and lingering during the acute phase, albeit in lower intensity and breadth. However, PD mice consistently displayed milder neurological deficit scores compared to CD mice.

### CSF1R signaling dictates the localization of inflammatory foci in the CNS in EAE

To address the seemingly conflicting data of increased CNS infiltration in PD mice but milder classical EAE clinical symptoms compared to CD mice, we initially hypothesized that the infiltrating cells may not efficiently penetrate the CNS of PD mice. To examine whether this was the case, we employed Evans blue dye (Eb) as we have previously described [40]. For this analysis, we employed mice within 24 h after the first symptom of EAE appeared in CD mice. At this timepoint, the majority of PD mice had no classical EAE symptoms. As expected, spinal cords of CD mice had extensive Eb-infiltrated areas. However, PD spinal cords showed minor Eb foci. In contrast, we observed increased Eb-infiltrated areas in the cerebellum of PD mice, but mild Eb presence in the cerebellum of CD mice (Additional file 6).

**Fig. 10:**
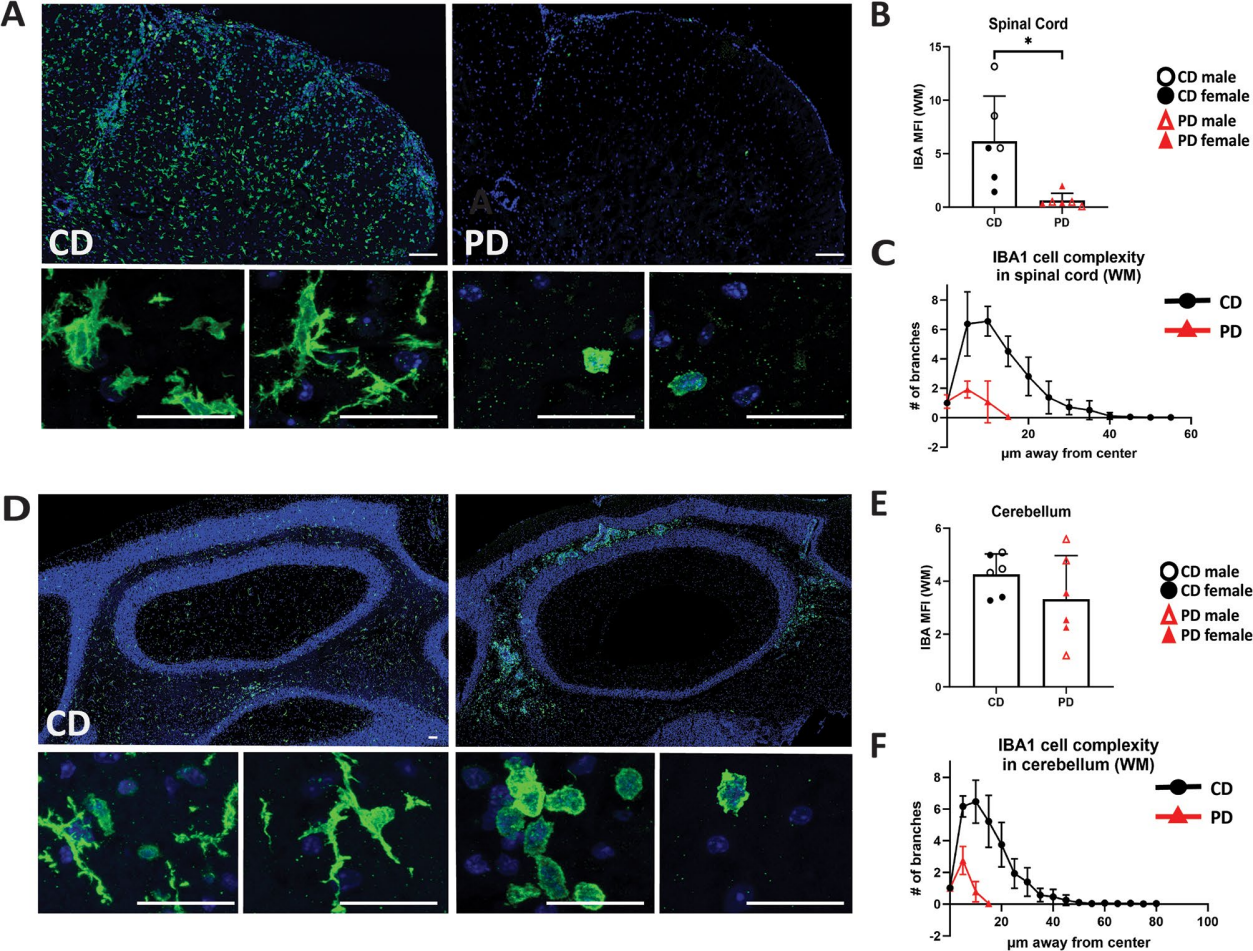
IBA1+ cells in the PD CNS of EAE mice have activated morphology and low complexity. CNS sections of CD and PD mice isolated during EAE onset were immunostained for IBA1. **A/D** Representative images of IBA1 immunoreactivity in lumbar spinal cords (**A**) and cerebellar (**D**) tissues of mice. **B/E.** Quantification of IBA1 MFI of lumbar spinal cord (**B**) and cerebellar (**E**) tissues of CD and PD mice. **C/F** Sholl analysis of IBA1+ cells near inflammatory foci shows low complexity and activated morphology in PD mice, but increased complexity in CD mice. (**C**: spinal cord; **F** cerebellum). Scale bars denote 100 μm in whole tissue images and 25 μm in enlarged cellular-level images. Data are shown as means ± SD, *n* = 6–7 mice; 2 sections per tissue; 4–8 microglia per section; *p < 0.05

At EAE onset, IBA1+ cells in the CD lumbar spinal cords were readily detected while as expected, they were largely absent from the EAE PD spinal cord (Fig. 10A and B) (CD: 6.17 vs. PD: 0.626; *p* = 0.01). IBA1+ cells in CD spinal cords displayed activated morphology (yet not ameboid) [55] maintaining some degree of branching, thick short processes, and larger somas (Fig. 10A; bottom left inserts). In the spinal cords of PD mice, there were few IBA1+ cells in the white matter, which exhibited low or no branching and an ameboid morphology, and were mostly located at the edge of the tissue (coinciding with the very few and small inflammatory foci), suggesting they are mostly monocyte-derived macrophages (Fig. 10A; bottom right inserts). Sholl analysis showed that IBA1+ cells in PD spinal cords exhibited lower levels of complexity compared with IBA1+ cells in CD spinal cords (Fig. 10C).

In the cerebellum, IBA1+ cells were readily detected in both CD and PD mice (Fig. 10D and E) (CD: 4.25 vs. PD: 3.32; *p* = 0.24) at EAE onset. In CD mice, however, IBA1+ cells exhibited higher degree of complexity and moderately activated morphology (Fig. 10D; bottom left insert). In PD cerebella, IBA1+ cells exhibited almost exclusively amoeboid morphology (Fig. 10D; bottom right insert), likely representing infiltrating cells that have migrated from the periphery within the tissue parenchyma. Thus, the increased IBA1 MFI in PD cerebella is due to infiltrating monocyte-derived macrophages, while IBA1 MFI in CD cerebella is the result of microglia and some infiltrating monocyte derived macrophages (Fig. 10E). Sholl analysis confirmed that the majority of CD cerebellar IBA1+ cells exhibited significantly higher levels of complexity and are presumably microglia (Fig. 10F).

**Fig. 11:**
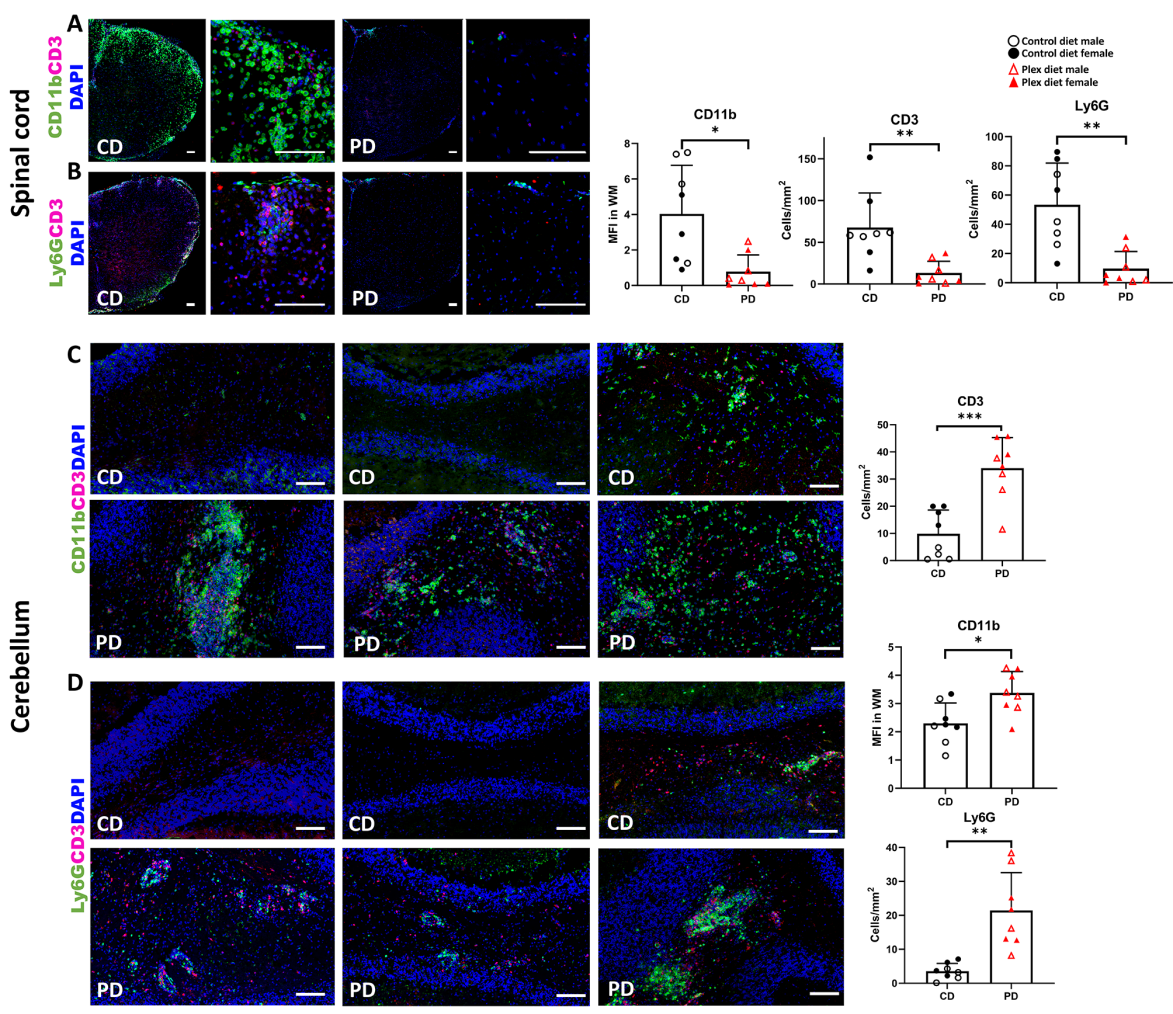
CNS infiltration is prominent in the cerebellum of PD mice with EAE. Lumbar spinal cords and cerebella were isolated at the onset of neurological deficits, and analyzed via immunohistochemistry for mean fluorescence intensity (MFI) of CD11b (myeloid cells), and for counts of CD3+ T cells and Ly6G+ neutrophils. **A/B** Spinal cords of CD mice show increased infiltration by myeloid cells, T cells (**A**), and neutrophils (**B**), while these cells are sparsely detected in the parenchyma or in the meninges of the PD spinal cords. Quantification is shown in graphs on the right. **C/D** Cerebella of CD mice are only mildly infiltrated by myeloid cells, T cells (**C**), and neutrophils (**D**), while these cells are found in large numbers in the cerebellum of PD mice. Quantification is shown in graphs on the right. Scale bars denote 100 μm. Both male and female mice were employed. Data are shown as means ± SD, *n* = 8; at least 2 sections per tissue; *p < 0.05, **p < 0.005, ***p < 0.0005

Accordingly, immunohistochemical analysis of lumbar spinal cords of CD EAE mice showed increased CD11b (all myeloid cells) MFI (Fig. 11A; graph on the right), and increased numbers of T cells (CD3+) (Fig. 11A; graph on far right), and of neutrophils (Ly6G+) (Fig. 11B; graph on right), compared to PD EAE spinal cords. Only a few cells were detected within the PD spinal cord parenchyma and meninges (Fig. 11A and B). This was confirmed via qPCR analysis showing that *Cd3e*, as well as *Cd4* and *Cd8a*, transcripts were increased in CD compared to PD spinal cords during EAE onset (Additional file 7A).

CD11b fluorescence intensity (Fig. 11C; graph on the right) and numbers of CD3+ T cells (Fig. 11C; graph on far right) and neutrophils, (Fig. 11D; graph on the right) were significantly increased in the cerebellum of PD compared to CD mice at EAE onset. Infiltration of cerebella was detected both in the perivascular cuffs and within the tissue parenchyma (Fig. 11C and D).

These data were confirmed via qPCR analysis showing that *Cd3e* transcripts were increased in PD cerebella compared to that of the CD group. *Cd8a* transcripts were also increased, but no difference was detected in *Cd4* transcripts in PD compared to CD cerebella (Additional file 7A). Although the increased *Cd4* transcripts in the CD group may be suggestive of CD4+ T cell infiltration, this increase is likely due to *Cd4* mRNA expression in microglia, which has been previously documented in numerous RNAseq studies [56–59].

All the above show that during EAE onset, infiltration was more prominent in the spinal cords of CD mice and in the cerebella of PD mice. However, one CD mouse, which acutely developed severe neurological deficits (typical EAE score of 3.5 from score 0 within 24 h; a rare event in our model), showed extreme infiltration in both the cerebellum and the spinal cord in levels that were higher than any other mouse in either group during this timepoint. This mouse was excluded from the analysis due to the abnormal disease pattern.

**Fig. 12:**
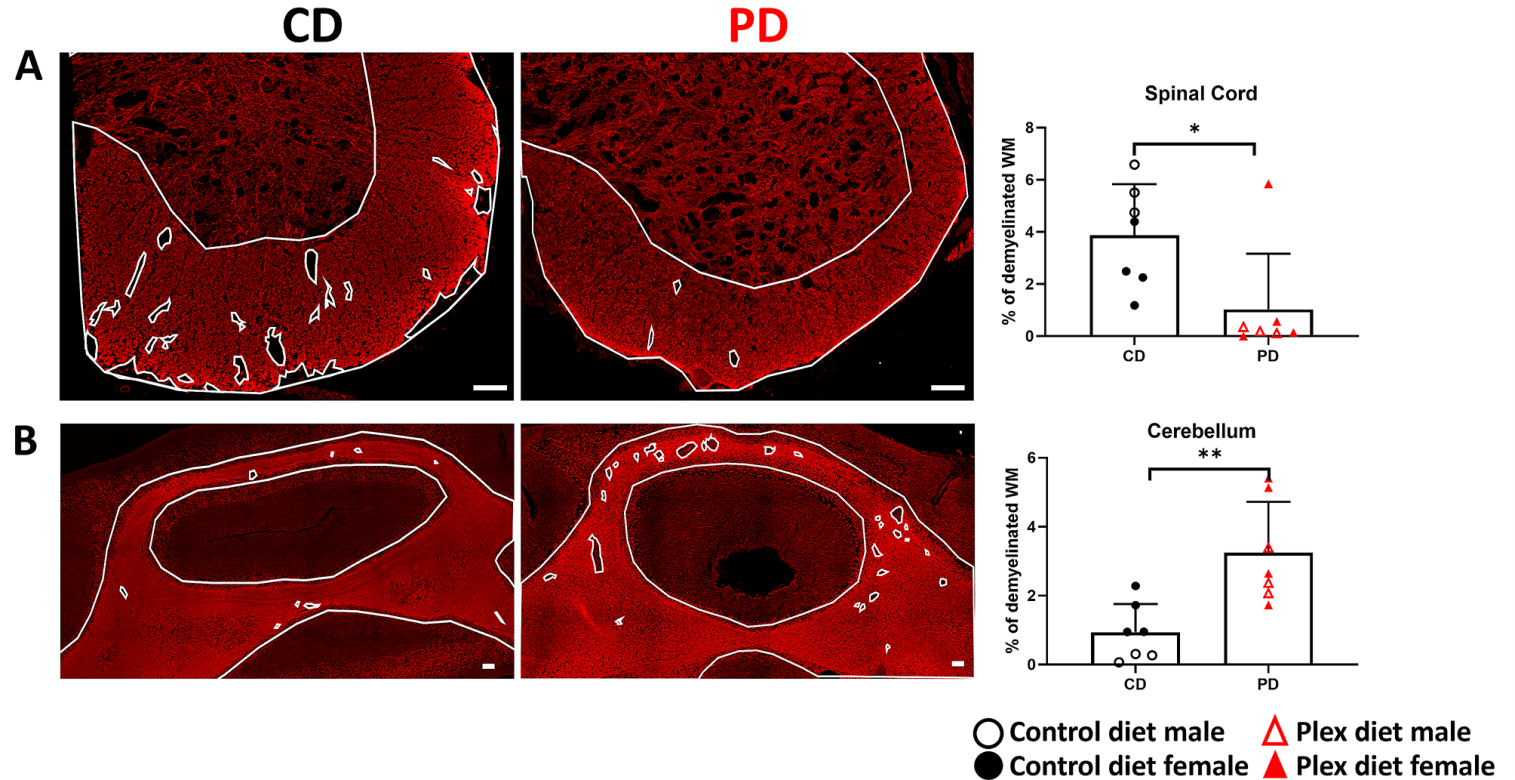
Increased demyelination in the cerebellum and decreased demyelination in the spinal cord of PD mice during EAE onset. Myelin was detected in lumbar spinal cord and cerebellar tissues isolated from mice during EAE onset via MBP immunostaining. Demyelinated areas (i.e., white matter devoid of MBP immunoreactivity; demarcated with small white borders) are readily detected in the spinal cords of CD but not in the spinal cords of PD (**A**) and cerebella of PD mice but not cerebella of CD mice (**B**). Quantification shows percentage of MBP devoid white matter (WM) with all WM area (graphs on the right). Scale bars denote 100 μm. Data are shown as means ± SD, *n* = 7; at least 2 sections per tissue; *p < 0.05, **p < 0.005

MBP immunoreactivity revealed increased demyelination in the spinal cord of CD compared to PD mice at EAE onset (Fig. 12A). On the contrary, there were significantly smaller demyelinated areas in the cerebellum of CD compared to PD mice (Fig. 12B).

All the above indicate that peripheral immune cells in PD mice enter the CNS but preferentially localize within the supraspinal regions, instead of lumbar spinal cords as is the case for the control mice.

### Effect of CSF1R antagonism in immune related transcripts in the CNS of mice with EAE

Our next step was to determine a possible mechanism underlying the preferential recruitment of peripheral immune cells into the cerebellum instead of the spinal cord of PD mice with EAE.

**Fig. 13:**
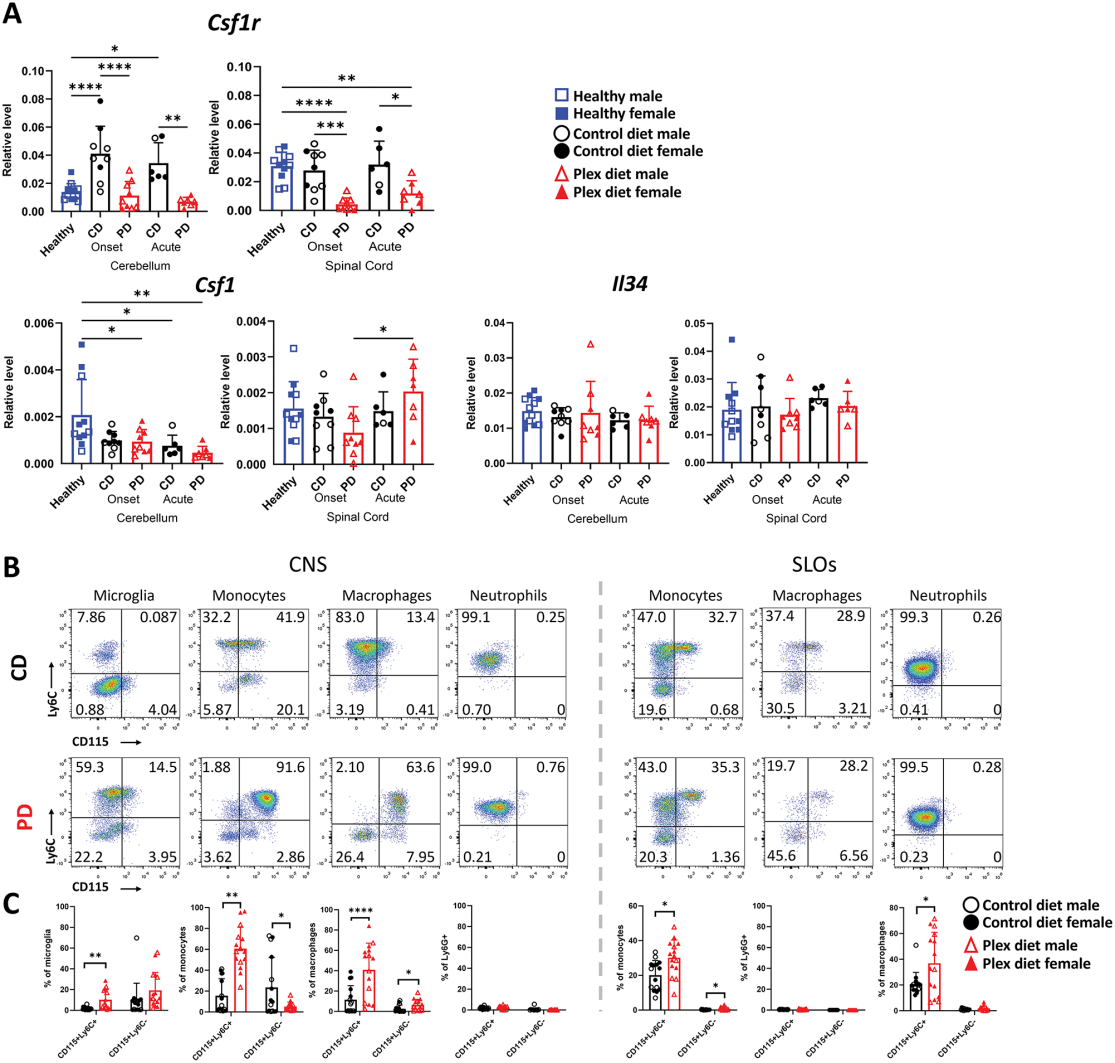
Effect of CSF1R antagonism on the expression of CSF1R and its ligands during EAE. **A** *Csf1r* transcript levels are lower in the PD compared to CD CNS during EAE. There were no differences in the transcripts of *Csf1* and *Il34*. **B** CSF1R (CD115) expression was detected using flow cytometry in Ly6C+ and Ly6C- microglia, monocytes, macrophages, and neutrophils in pooled brain and spinal cord (CNS), and SLOs during EAE. **C** Quantification of B. Data are shown as means ± SD, A: *n* = 9–12; B: *n* = 16 *p < 0.05, **p < 0.005, ***p < 0.0005, ****p < 0.00005

We initially examined whether the relative CSF1R levels are affected by PD. CNS *Csf1r* transcripts were significantly decreased in the PD compared to the CD group (Fig. 13A). This downregulation is likely due to the depletion of CSF1R-expressing microglia and other myeloid cell subsets. Interestingly, *Csf1r* levels in the cerebellum of CD mice were significantly upregulated compared to healthy tissue during both onset and acute EAE phases, but not in the spinal cord (Fig. 13A). It is likely that this differential *Csf1r* upregulation may reflect a compartment-dependent modulation of microglia activity. The expression of the CSF1R ligands *Csf1* and *Il34* in the CNS were not different between CD and PD mice (Fig. 13A).

Flow cytometric analysis showed that the remaining/surviving microglia and infiltrating monocytes/macrophages express high levels of CSF1R (CD115) in the CNS, but to a lesser degree in the periphery of PD compared to CD mice (Fig. 13B and C). CSF1R signaling is required for survival of macrophages [60] and of a Ly6C- subset of monocytes [37], and the differentiation of monocytes to macrophages [61].

Despite this dramatic increase of CSF1R expression in CNS infiltrating monocytes and macrophages in PD mice, this was not sufficient to increase *Csf1r* levels in the CNS to similar levels as those of CD CNS tissues (Fig. 13A). In addition, a caveat of flow cytometric CSF1R detection is that the CSF1R (CD115) antibody binding may be inhibited by CSF1R natural ligands [2], which may result in impaired detection of CSF1R in cells isolated from CD tissues.

Transcripts of *Csf1r* have been previously detected in neutrophils [62], however, we did not detect CSF1R expression in neutrophils by flow cytometry (Fig. 13B and C).

**Fig. 14:**
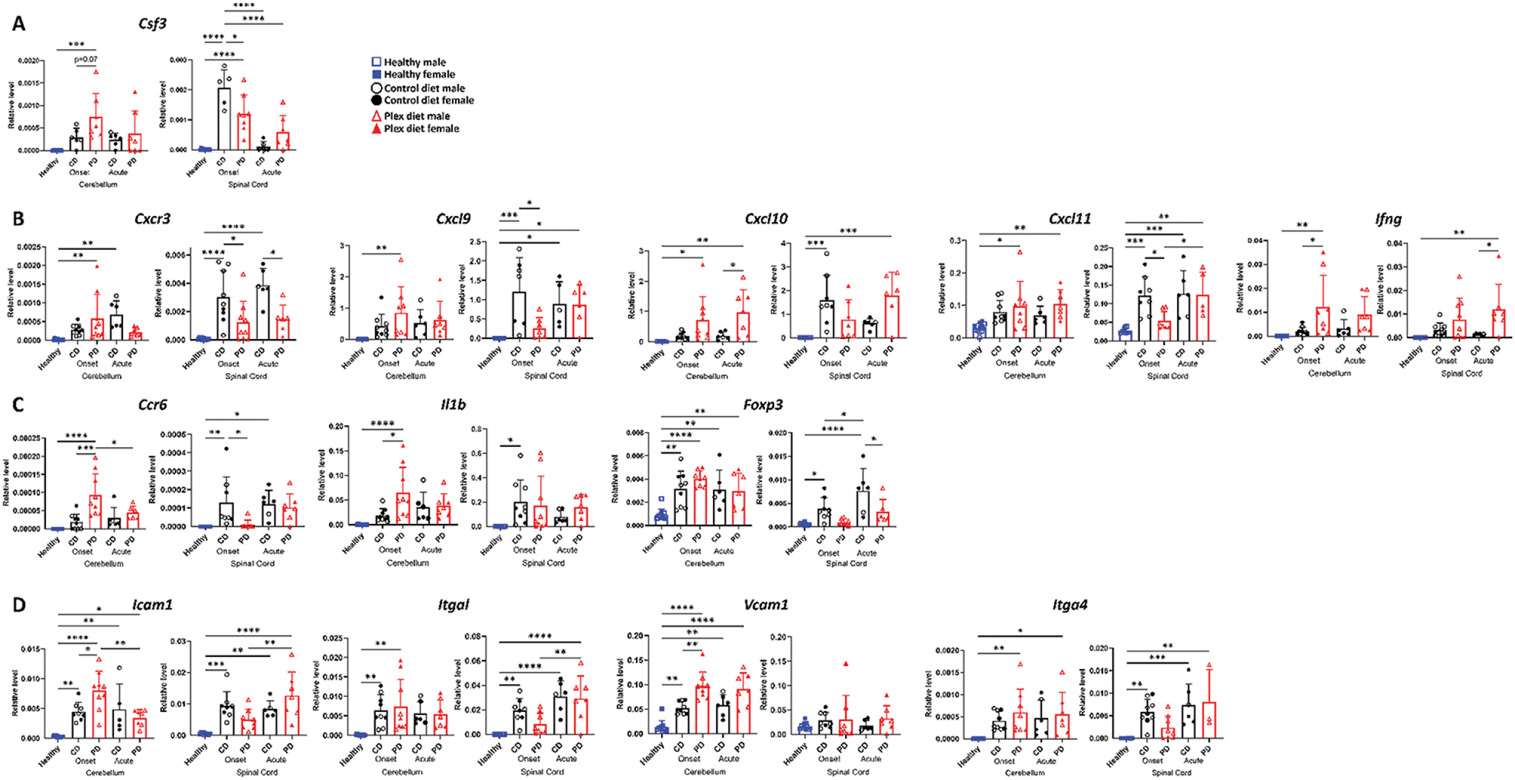
CSF1R antagonism differentially affects gene expression in CNS compartments. **A-D** Cerebella and spinal cords were isolated from CD and PD mice at onset and acute EAE and RNA transcripts were analyzed by quantitative PCR. Data are shown as means ± SD, *n* = 6–12; *p < 0.05, **p < 0.005, ***p < 0.0005, ****p < 0.00005

We then examined whether we could detect regional differences in the levels of transcripts of cytokines, chemokines, and transcription factors associated with EAE and MS [63, 64] (Fig. 14). We employed the whole cerebellum, a tissue which even when heavily infiltrated, contains large areas devoid of infiltration; thus, the signal of certain transcripts may be “diluted”. On the other hand, lumbar EAE spinal cord tissues contain heavily lesioned areas, thus the contribution of transcripts from unaffected tissue areas is small.

Interestingly, *Csf3*, encoding for G-CSF, was elevated in the PD cerebellum and CD spinal cord during disease onset (Fig. 14A). G-CSF has been previously shown to be expressed in the CNS around the time of clinical disease onset, and is associated with neutrophilic infiltration [43, 65] and neutrophilic survival [66]. These suggest that increased cerebellar *Csf3* transcripts promote preferential cerebellar neutrophilic infiltration in PD mice.

During disease onset, transcripts of *Cxcr3* and of its ligands *Cxcl9* and *Cxcl11* were decreased in the PD spinal cords, suggesting a decrease in Th1-associated chemokines compared to CD (Fig. 14B). Interestingly, the levels of *Cxcl10* and *Ifng* (genes encoding for Th1-associated responses)  were elevated in the cerebellum of PD mice. In addition, CSF1R antagonism resulted in statistically significant transcript increases of *Il1b* and *Ccr6* (a receptor expressed on both Th17 and Tregs [67–69]) in the cerebellum of PD compared to CD mice (Fig. 14C).

*Icam1*, which is heavily involved with leukocyte cell arrest, subsequent diapedesis, polarization and crawling across the BBB [70–72], and *Vcam1*, which is involved with Th1 and Th17 cell arrest [73], were both elevated in the cerebellum of PD mice but not in their spinal cords compared to CD mice during EAE onset (Fig. 14D). Although their ligands (*Itgal* and *Itga4*, respectively) were not differentially upregulated between the groups (Fig. 14D), the differences in *Icam1* and *Vcam1* expression suggest that these molecules may orchestrate the localization of CNS infiltrates in EAE.

Additional transcripts of genes associated with EAE induction and progression were tested but did not show differences in expression between the two groups during disease onset (Additional file 7B).

## Discussion

CSF1R antagonists were originally developed to deplete tumor-associated macrophages, which promote tumor growth, survival, angiogenesis, treatment resistance, and metastasis [74–76]. Tumors treated with CSF1R antagonists are infiltrated by increased CD4+ T cells, pro-inflammatory macrophages, and other pro-inflammatory leukocytes [21, 22, 77]. As a result, administration of these antagonists as part of combination therapy enhances the efficacy of anti-cancer therapeutics manifold [77].

Along with other tissue macrophages, small molecule CSF1R antagonists can also effectively deplete microglia [32], and have been shown to ameliorate EAE clinical course [16, 17, 35]. The mechanism of action is still nebulous. Studies have shown that the dampening of EAE clinical symptoms in mice treated with CSF1R antagonists is associated with depletion of microglia and CNS-associated macrophages, as well as decreased CNS infiltration and peripheral myeloid cell maturation [16, 17, 35].

Using PLX5622 diet (PD) and in agreement with previous studies [16, 35], we also observed efficient microglial depletion and milder EAE clinical course in PD mice compared to controls. However, and contrary to other studies [16, 17, 35], we show that PD mice exhibited increased CNS infiltration as documented by increased presence of myeloid subsets, including inflammatory monocytes, neutrophils, and cDCs, and T cell subsets (both T effectors and Tregs) compared to controls (CD) before and during EAE onset.

Interestingly, we observed that during disease onset spinal cords of PD mice were largely devoid of infiltration, while their cerebella were severely infiltrated. As expected, at the same timepoint, infiltration was mainly localized in the spinal cords of controls. Accordingly, larger demyelinated areas were detected in the spinal cords of CD compared to PD mice, while increased demyelination was detected in the cerebella of PD compared to CD mice.

Unlike damage to the spinal cord, which presents with motor deficits [78], cerebellar infiltration manifests in deficits in cognitive, emotional, and behavioral function, induction of fear and anxiety [79, 80], and impairments in balance and proprioception [81–84]. We observed that one third of EAE PD mice developed seizures, exhibited hypersensitivity for startle response, freezing behavior, and repetitive axial rotation. At the time of atypical EAE onset, PD mice exhibited mild or no classical EAE symptoms. Furthermore, although we detected some cerebellar infiltration in CD mice, these mice never developed atypical EAE symptoms. Additionally, a subset of PD mice died unexpectedly early in the disease course, suggesting that CSF1R antagonism can be detrimental in neuroinflammation. No deaths were observed in CD mice. 

Our data are in apparent disagreement with previous studies by Montilla et al. [35], who also used PLX5622 and Hwang et al. [17], who used BLZ945, a CSF1R antagonist that acts similarly to PLX5622 [32]. BLZ945 and PLX5622 have similar potency (both have an IC_50_ < 10nM) [85–87] and CNS penetration properties [17, 35]. Both of the previous studies showed decreased infiltration of the CNS in mice that were treated with CSF1R antagonists. However, Montilla et al. examined only spinal cords, which we show were not effectively infiltrated in PD mice. In contrast, we used pooled brains and spinal cords for our flow cytometry analysis. Although Hwang et al. also used pooled brains and spinal cords, they documented dramatically lower numbers of infiltrating cells in mice that received BLZ945.

We also observed mild differences between our study and the previous studies in peripheral immune responses. In addition to decreases in macrophage populations, we show that PD mice exhibited decreased numbers of DCs in the SLOs during preclinical EAE, as shown by Hwang et al. [17]. Although we detected increased neutrophil and monocyte frequencies in PD SLOs, these did not translate to differences in absolute numbers, in agreement with Montilla et al., but in disagreement with Hwang et al. Furthermore, Montilla et al. and Hwang et al. did not detect differences in total peripheral CD4+ T cells but no information on CD4+ T cell subsets was provided [17, 35]. In agreement with these studies, we did not detect differences in total peripheral CD4+ T cell numbers either. However, we observed significant decreases in both frequencies and absolute numbers of T effectors and T regulatory cells in the SLOs of PD mice compared with controls during preclinical EAE. This suggests that in the absence of antigen presenting cells (which are depleted in the PD mice), T cells did not differentiate effectively.

One of the reasons for the discrepancies between our study and the Hwang et al. study could be that they initiated CSF1R blockade on the day of EAE induction, while we did so seven days earlier, better approximating the Montilla study in which treatment was initiated three weeks before EAE induction. It is likely that in the Hwang study there was not enough time for myeloid cells to adapt to the CSF1R blockade both in the SLOs and after these enter the CNS. Indeed, the Hwang study shows that mice treated with BLZ945 starting on day 0 eventually developed clinical deficits during the later EAE stages, however no information on the make-up and numbers of CNS infiltrating cells was provided.

Interestingly, immunohistological analysis of PD lumbar spinal cords at later stages of the disease (day 37 post EAE induction, which corresponds to day 44 on PD) showed increased microglial presence compared to preclinical and acute EAE stages (Additional file 8). After depletion, microglia repopulation occurs via proliferation of surviving/remaining microglia [88]. In inflammatory conditions peripheral myeloid cells may give rise to microglia-like cells that are phenotypically similar but transcriptionally different than microglia, and may not survive long-term [89–91]. In this study, repopulation occurred while the mice were still on PD, suggesting that the newly appearing microglia derived via a CSF1R- independent mechanism. Whether the repopulation occurs from PD surviving microglia or residual infiltrating monocyte-derived macrophages is still unclear.

The Hwang et al. study [17] further showed that treatment initiation after clinical symptom onset ameliorated the disease progression for the following 7 or 14 days, but there is no information on whether the mice relapsed later (i.e., after the mice were at least three weeks on the treatment). Interestingly, we did not detect differences in the clinical scores when mice were placed on PD after clinical symptoms appeared (data not shown).

Other factors that impact disease development and progression of EAE include differences in the environment, such as microbiota and mouse husbandry, and in the formulation of immunization reagents.

The mechanism(s) behind preferential recruitment of peripheral cells to the cerebellum instead of the spinal cord in PD mice remain to be elucidated. Increases in neutrophil frequencies, an indirect effect of prolonged CSF1R blockade, both in the periphery and the CNS of PD mice compared to controls, are associated with preferential infiltration of the supraspinal regions and with atypical EAE [92–94]. Previous studies have shown that CSF1R blockade increases G-CSF levels [95], a cytokine responsible for the survival, proliferation, maturation, and function of neutrophils [96]. Indeed, *Csf3* was elevated in the PD cerebellum and diminished in the PD spinal cords during the onset of clinical symptoms. Neutrophils are also known to recruit other leukocytes, especially inflammatory monocytes, to sites of inflammation by upregulating cell adhesion molecules such as ICAM1 and VCAM1, and secreting chemokines such as CXCL9 and CXCL10 [97, 98]. Indeed, we detected elevation of *Icam1, Vcam1 and Cxcl10* transcripts in the cerebellum of PD mice at EAE onset. Increased neutrophil frequencies may have promoted the recruitment of increased numbers of monocytes and other immune cells detected in the cerebellum in our study.

A complementary explanation stems from the study by Stromnes et al. [99], which shows that CNS infiltrates in mice with EAE exhibiting a log10 ratio of Th17:Th1 > 1 in the periphery tend to localize in supraspinal areas, while in EAE mice with a log10 ratio of Th17:Th1 < 1 CNS infiltrates tend to localize in the spinal cord [99]. These ratios were also confirmed in the brains and spinal cords of mice with EAE [99]. We also show that T cells in the SLOs of PD mice during the preclinical stage of EAE had log10 ratios of Th17:Th1 higher than 1, which could partly explain the changes in lesion localization. The increase in Th17:Th1 ratios may be related to the PD-mediated depletion of Th1-promoting cells in peripheral tissues such as moDCs [100–103], or skin langerin+ DCs [104].

Our analysis of the effects of CSF1R antagonism on myeloid and lymphoid compartments extends current understanding about CSF1R effects in neuroinflammation. Although FDA-approved CSF1R antagonists do not penetrate the CNS as effectively as PLX5622, their prolonged use may be detrimental. The pleiotropism of CSF1R signaling likely has other yet undiscovered effects both in steady state and in disease settings that warrant further investigations.

### Electronic supplementary material

Below is the link to the electronic supplementary material.

**Additional file 1: Effect of CSF1R antagonism on CNS immune cell populations in steady state. A** Microglia are depleted efficiently in the PD spinal cord and cerebellum. **B** Effect of PD on microglia and CNS-associated myeloid cells and lymphocytes in whole CNS tissues (pooled brain and spinal cord per mouse) in PD and CD steady state mice. Data are shown as means ± SD, n = 7; *p < 0.05, **p < 0.005, ***p < 0.0005

**Additional file 2: Clinical EAE course of individual experiments.** Mice were placed in CD or PD diets seven days before EAE induction and maintained in their respective diets up to the end of the experiment. Mice were scored daily for neurological deficits. All experiments except experiment 6 were averaged to generate the clinical score graph in Figure 3. Data are shown as means ± SEM, n values are displayed within each chart; *p < 0.05

**Additional file 3: Increased neutrophils and inflammatory monocytes persist in the SLOs of PD mice during acute EAE. A-C** Single cell suspensions of pooled spleen and draining lymph nodes were analyzed for myeloid (B) or lymphocytic populations (C) during acute EAE. Both frequencies and numbers of neutrophils and Ly6C+ monocytes were elevated in the SLOs of PD compared to CD mice. No other differences were detected. Data are shown as means ± SD, n = 12; *p<0.05, **p<0.005

**Additional file 4: CSF1R antagonism affects cellular composition in the bone marrow during EAE.** Flow cytometric analysis of the bone marrow myeloid cell subsets in PD and CD mice before clinical symptoms (A) and during acute EAE (B). Data are shown as means ± SD, n = 8; *p < 0.05, **p < 0.005, ***p < 0.0005, ****p < 0.00005

**Additional file 5: CSF1R antagonism depletes antigen presenting cells and other myeloid subsets in the skin during EAE. A** Flow cytometric analysis of skin myeloid cell subsets in PD and CD mice before clinical symptoms (preonset) and **B** during acute EAE show that langerin+ DCs are dramatically reduced in PD mice. **C** Immunohistological analysis showed that CD11b+ cells and IBA1+ cells, which are all myeloid cells and macrophages, respectively, are reduced in the skin of PD compared to CD mice. Scale bars denote 25µm. Data are shown as means ± SD, n = 8, *p ± 0.05, ****p ± 0.00005

**Additional file 6. Mapping areas of CNS infiltration using Evans blue dye.** Evans blue dye was injected intravenously into CD and PD mice with EAE. Ninety minutes later, mice were euthanized, and tissue was isolated. Dorsal and ventral images were acquired on day 13 EAE (onset) in CD and PD mice

**Additional file 7: RNA transcript levels in the CNS compartments of CD and PD mice during EAE.** Cerebella and spinal cords were isolated from CD and PD mice during steady state, and clinical onset and acute EAE. RNA transcripts were measured using qPCR analysis. Data are shown as means ± SD, n = 6–12; *p < 0.05, **p < 0.005, ***p < 0.0005, ****p < 0.00005

**Additional file 8. Repopulation of lumbar spinal cords of PD mice by microglia during chronic EAE.** **A** Spinal cord of CD and PD mice were isolated during different stages of EAE and immunostained with IBA1. **B** quantification of **(A)** shows IBA1 MFI increases over time in the PD spinal cord. These mice were still maintained in PLX5622 diet. It is unclear whether these are cells that originate from a local progenitor or an infiltrating monocytic cell

## Data Availability

All data in this study are included in this published article and its additional files.
