# Supplementary figures and images for: CSF1R antagonism results in increased supraspinal infiltration in EAE
Source: J Neuroinflammation. 2024 Apr 20;21:103. doi: 10.1186/s12974-024-03063-1 (PMC11031888; doi:10.1186/s12974-024-03063-1)

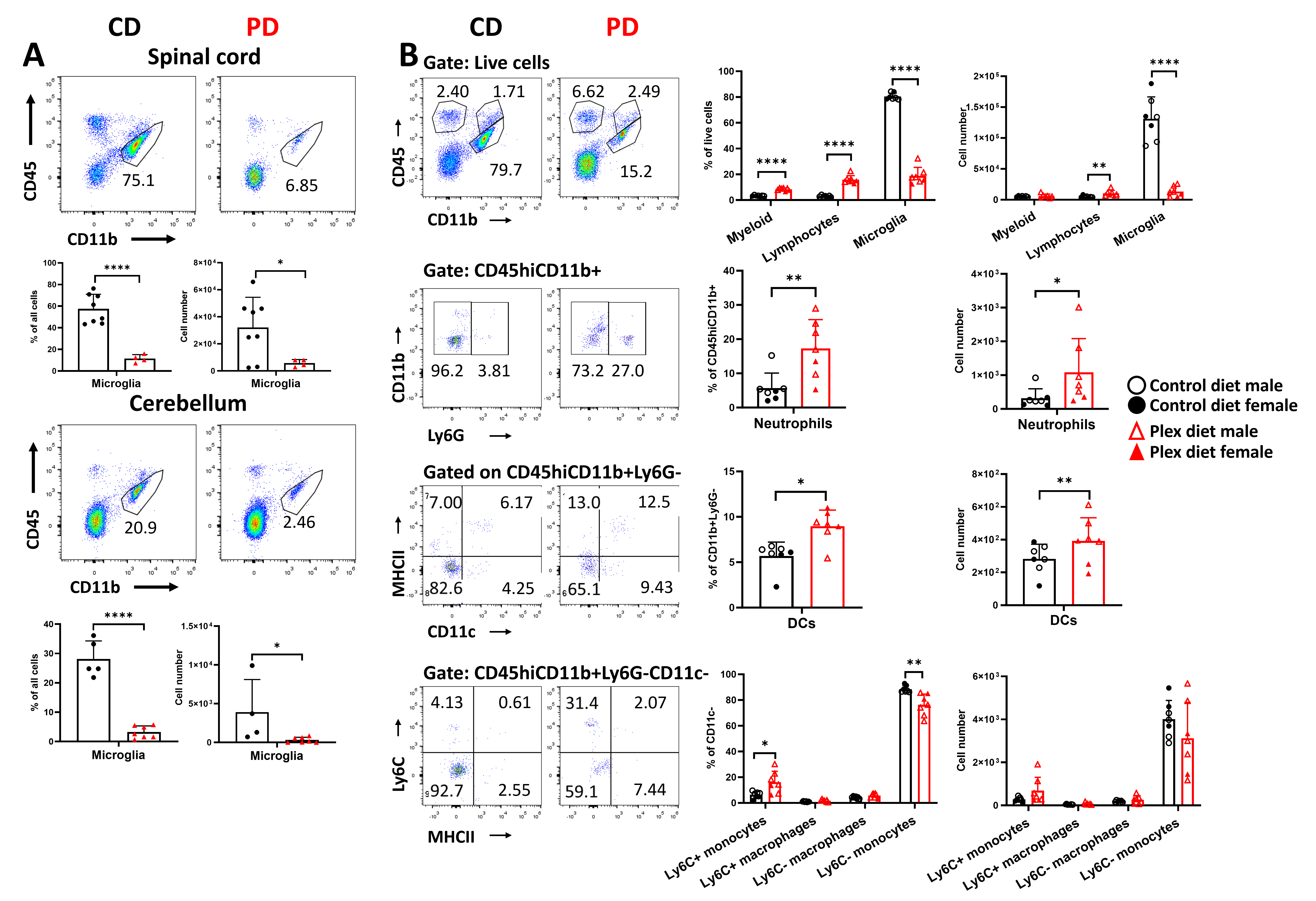

Supplement: Supplementary file 1 — Additional file 1: Effect of CSF1R antagonism on CNS immune cell populations in steady state. A Microglia are depleted efficiently in the PD spinal cord and cerebellum. B Effect of PD on microglia and CNS-associated myeloid cells and lymphocytes in whole CNS tissues (pooled brain and spinal cord per mouse) in PD and CD steady state mice. Data are shown as means ± SD, n = 7; *p < 0.05, **p < 0.005, ***p < 0.0005 [file 12974_2024_3063_MOESM1_ESM.tif]

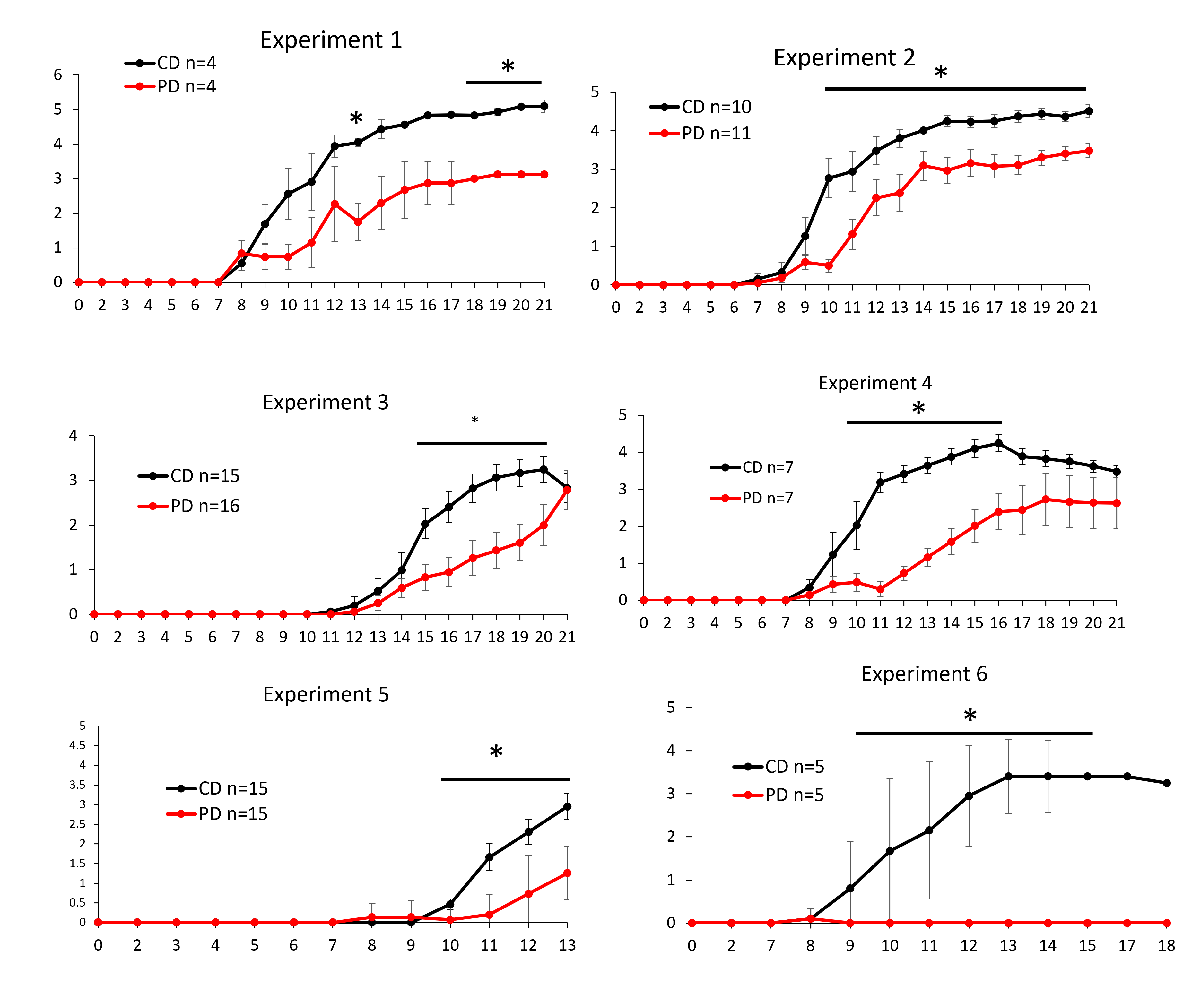

Supplement: Supplementary file 2 — Additional file 2: Clinical EAE course of individual experiments. Mice were placed in CD or PD diets seven days before EAE induction and maintained in their respective diets up to the end of the experiment. Mice were scored daily for neurological deficits. All experiments except experiment 6 were averaged to generate the clinical score graph in Figure 3. Data are shown as means ± SEM, n values are displayed within each chart; *p < 0.05 [file 12974_2024_3063_MOESM2_ESM.tif]

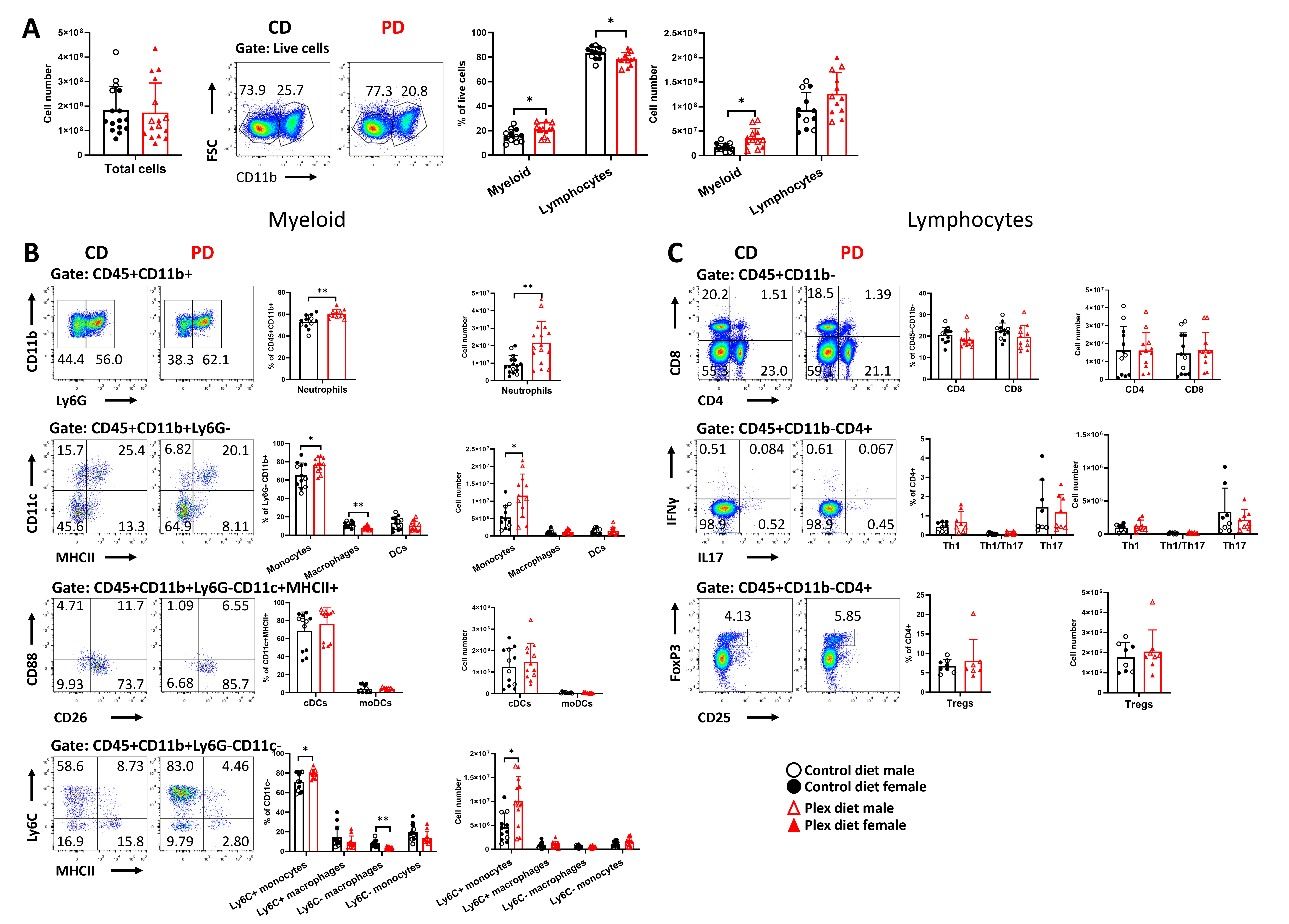

Supplement: Supplementary file 3 — Additional file 3: Increased neutrophils and inflammatory monocytes persist in the SLOs of PD mice during acute EAE. A-C Single cell suspensions of pooled spleen and draining lymph nodes were analyzed for myeloid (B) or lymphocytic populations (C) during acute EAE. Both frequencies and numbers of neutrophils and Ly6C+ monocytes were elevated in the SLOs of PD compared to CD mice. No other differences were detected. Data are shown as means ± SD, n = 12; *p<0.05, **p<0.005 [file 12974_2024_3063_MOESM3_ESM.tif]

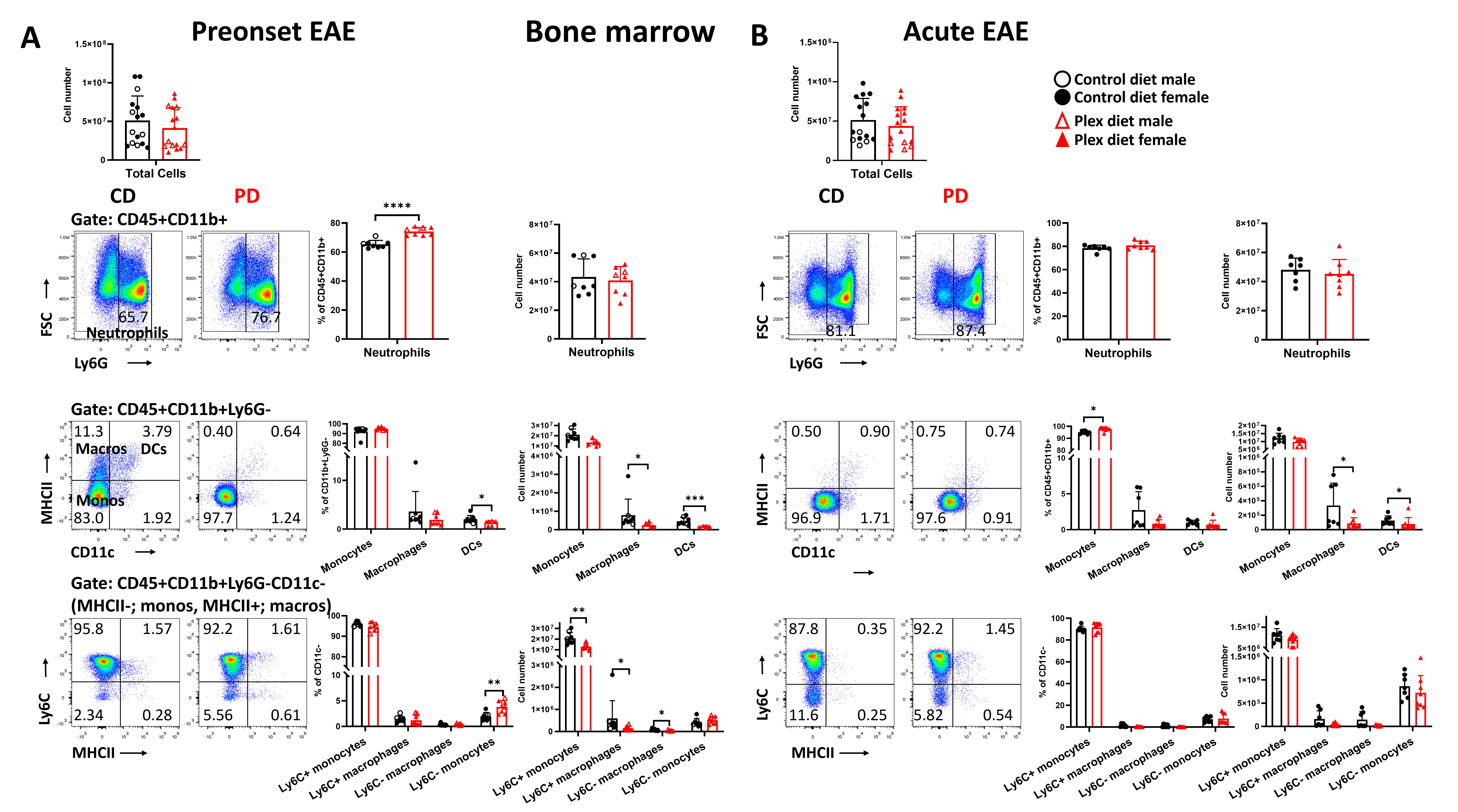

Supplement: Supplementary file 4 — Additional file 4: CSF1R antagonism affects cellular composition in the bone marrow during EAE. Flow cytometric analysis of the bone marrow myeloid cell subsets in PD and CD mice before clinical symptoms (A) and during acute EAE (B). Data are shown as means ± SD, n = 8; *p < 0.05, **p < 0.005, ***p < 0.0005, ****p < 0.00005 [file 12974_2024_3063_MOESM4_ESM.tif]

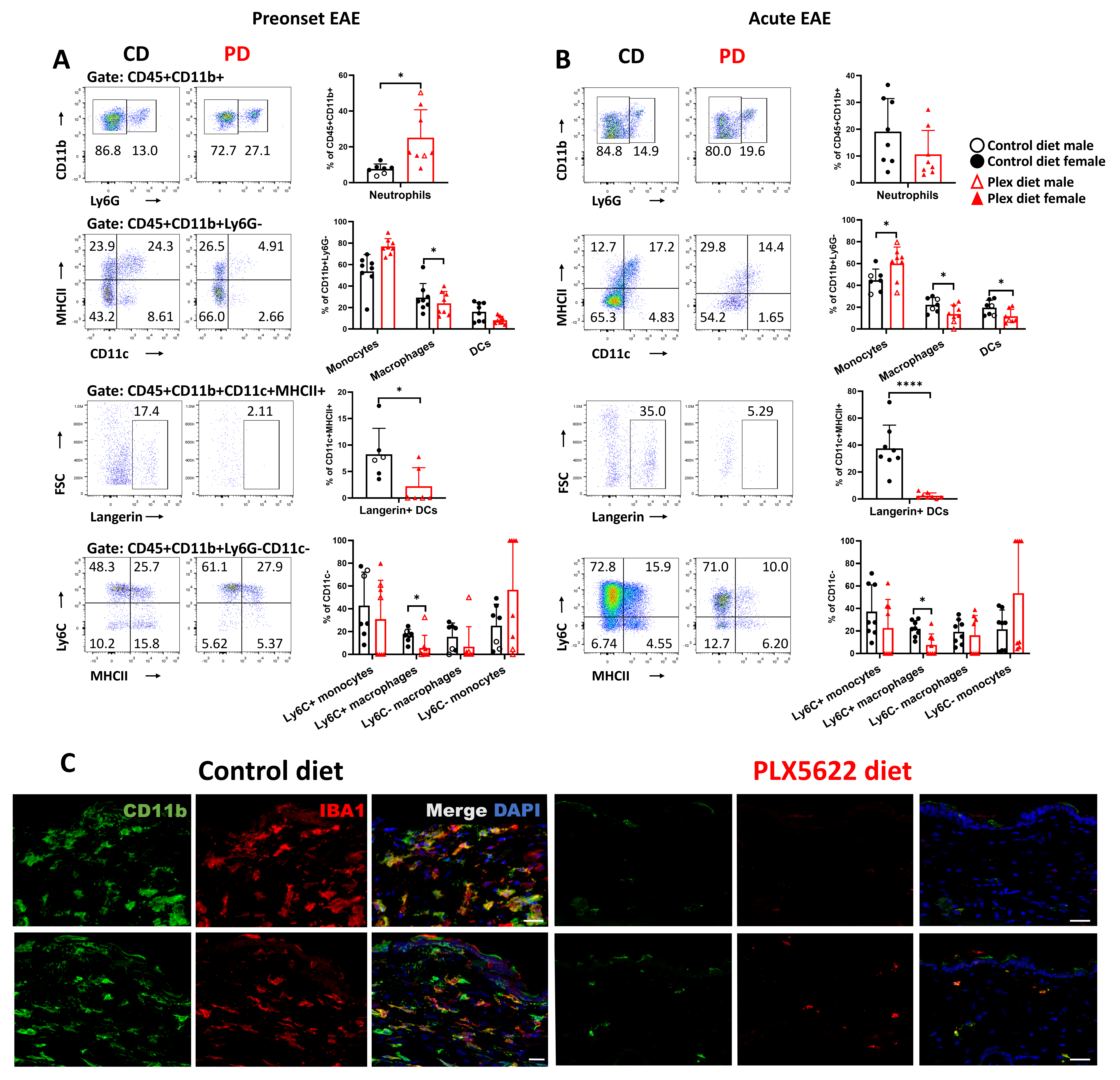

Supplement: Supplementary file 5 — Additional file 5: CSF1R antagonism depletes antigen presenting cells and other myeloid subsets in the skin during EAE. A Flow cytometric analysis of skin myeloid cell subsets in PD and CD mice before clinical symptoms (preonset) and B during acute EAE show that langerin+ DCs are dramatically reduced in PD mice. C Immunohistological analysis showed that CD11b+ cells and IBA1+ cells, which are all myeloid cells and macrophages, respectively, are reduced in the skin of PD compared to CD mice. Scale bars denote 25µm. Data are shown as means ± SD, n = 8, *p ± 0.05, ****p ± 0.00005 [file 12974_2024_3063_MOESM5_ESM.tif]

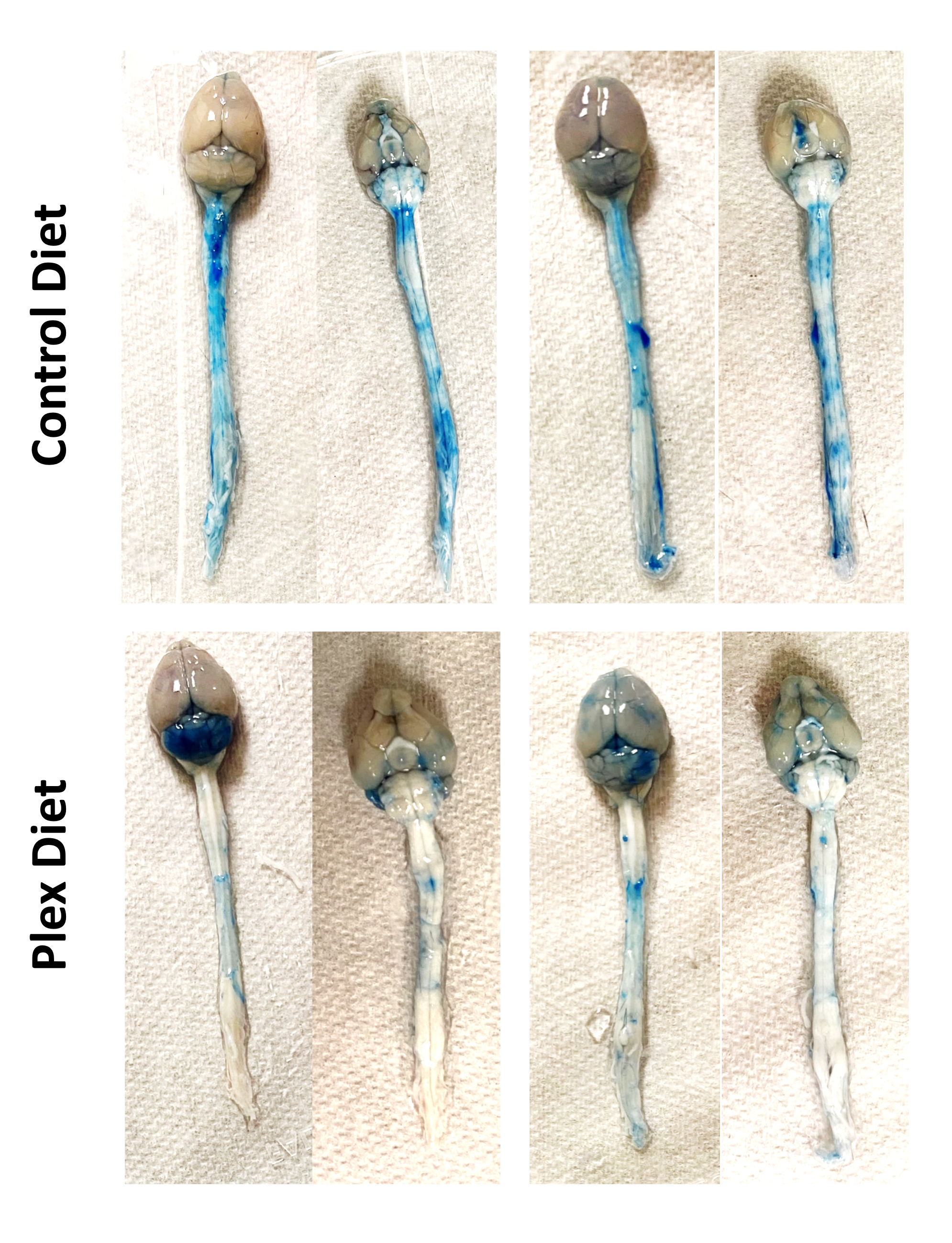

Supplement: Supplementary file 6 — Additional file 6. Mapping areas of CNS infiltration using Evans blue dye. Evans blue dye was injected intravenously into CD and PD mice with EAE. Ninety minutes later, mice were euthanized, and tissue was isolated. Dorsal and ventral images were acquired on day 13 EAE (onset) in CD and PD mice [file 12974_2024_3063_MOESM6_ESM.tif]

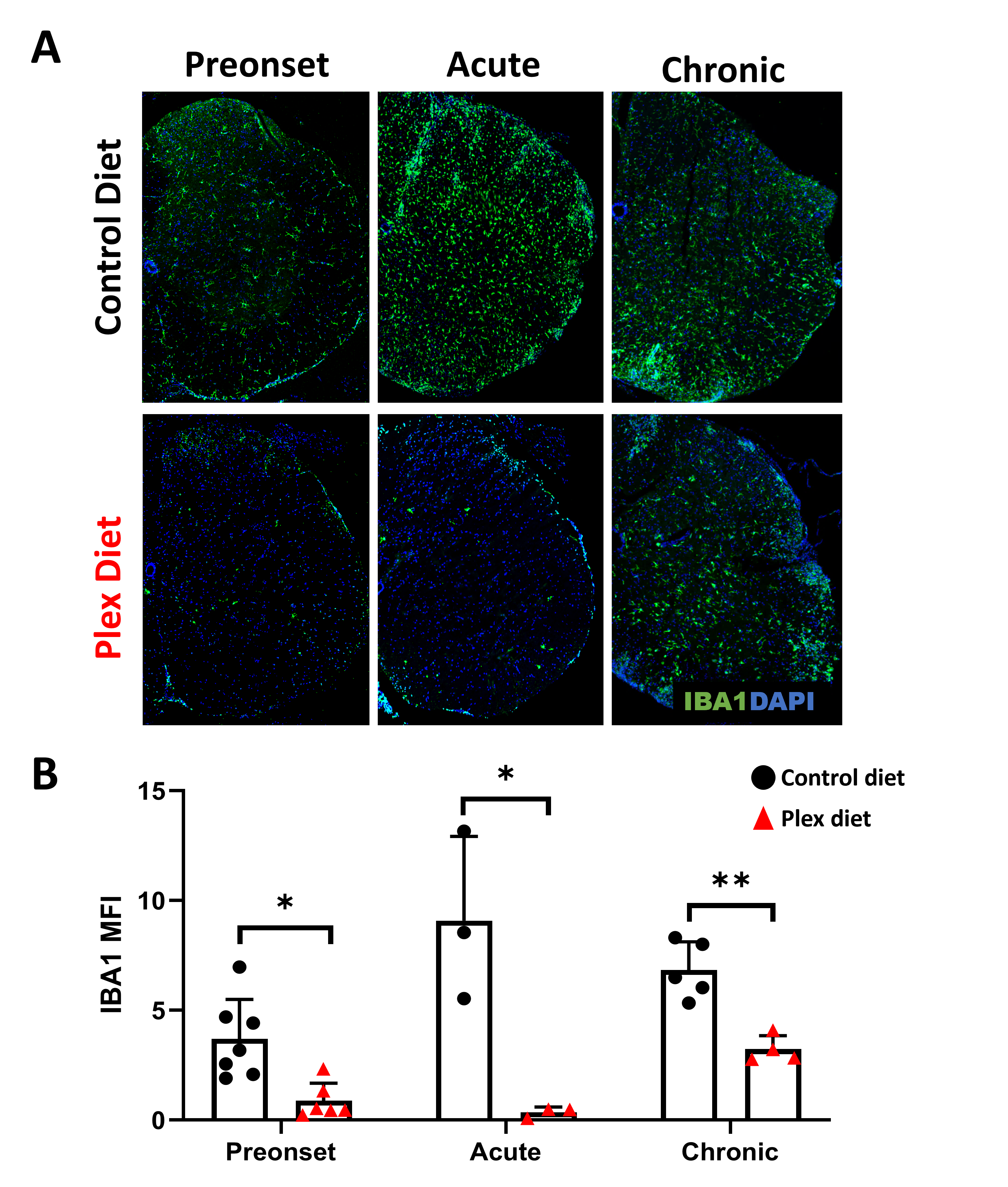

Supplement: Supplementary file 8 — Additional file 8. Repopulation of lumbar spinal cords of PD mice by microglia during chronic EAE. A Spinal cord of CD and PD mice were isolated during different stages of EAE and immunostained with IBA1. B quantification of (A) shows IBA1 MFI increases over time in the PD spinal cord. These mice were still maintained in PLX5622 diet. It is unclear whether these are cells that originate from a local progenitor or an infiltrating monocytic cell [file 12974_2024_3063_MOESM8_ESM.tif]
